# Supplementary material for: Transcriptomic classes of BCR-ABL1 lymphoblastic leukemia
Source: Nat Genet. 2023 Jun 19;55(7):1186–97. doi: 10.1038/s41588-023-01429-4 (PMC10335939; doi:10.1038/s41588-023-01429-4)
Supplement: Supplementary file 1 — Supplementary Note, Supplementary Figs. 1–35 and Supplementary References. [file 41588_2023_1429_MOESM1_ESM.pdf]

nature genetics

Article

https://doi.org/10.1038/s41588-023-01429-4

Transcriptomic classes of *BCR-ABL1* lymphoblastic leukemia

In the format provided by the authors and unedited

Transcriptomic classes of *BCR-ABL1* lymphoblastic leukemia

SUPPLEMENTARY INFORMATION

Supplementary results

Supplementary figures

Supplementary references

Supplementary results

Unique immunophenotypic patterns of the molecular subtypes

We assessed the flow cytometry patterns of CD34 and CD19 antigens that were used to identify and sort blasts. Although clinical immunophenotyping reported positivity of CD34 and CD19 on nearly all leukemias in the cohort, we observed six distinct profiles of CD34 and CD19 expressions on blasts (Fig. S26a). Strikingly, these six blast types were unevenly distributed across the three subtypes. Early-Pro was enriched for blast types 2 and 3, which expressed dim to low levels of CD19; Inter-Pro was enriched for blast type 1, which expressed high levels of both CD34 and CD19; and Late-Pro was enriched for blast types 4 and 5, which expressed dim to low levels of CD34 (p=1.8e-11, Fisher's exact test; Fig. S26b). Consequently, we observed greater proportions of $CD34^{+}CD19^{-}$ cells and $CD34^{-}CD19^{+}$ cells in Early-Pro and Late-Pro leukemias, respectively (FDR-adjusted p=1.1e-5 and 0.0063, Kruskal-Wallis test; Fig. S27). Ph50-D (C1/Early-Pro) was the only leukemia with blast type 6, which lacks CD34 expression (Fig. S26a, bottom right panel). In summary, the three molecular subtypes of BCR-ABL1 lymphoblastic leukemia display distinct patterns antigen expressions.

Rearrangement patterns of the immunoglobulin heavy chain locus

We aimed to determine whether leukemic phenotypes are maintained from the cell type in which they are arrested in or acquired during the transformation process. To tackle this question, we examined immunoglobulin heavy chain locus (*IGH@*) rearrangements in the RNA-seq and WGS data. Legitimate and illegitimate rearrangements of *IGH@* are almost invariably observed in *BCR-ABL1* ALL, B/myeloid MPAL, and CML-LBC[92–94]. In agreement with those reports, all leukemias in our cohort carried rearranged *IGH@* although most of the resulting V(D)J sequences were non-productive (Fig. S10b).

Since the *IGH@* contracts as B-cells mature from early to late stages to promote utilization of distal V_H genes[26,95,96], we hypothesized that leukemias arising from an early developmental stage would preferentially show short-range rearrangements with proximal V_H segments while those arising from a later stage would show long-range rearrangements utilizing distal V_H segments (Fig. S28a). When *IGH@* rearrangements were compared across the subtypes, significantly greater proportions of rightside breakpoints in Inter-Pro and Late-Pro leukemias were located in the distal V_H region than those in Early-Pro leukemias (Fig. S28a; p=0.042, Kruskal-Wallis test). Using the mclust algorithm[97], *IGH@* breakpoints were clustered into 5 bins (Fig. S28b), and similarly, breakpoints in Inter-Pro and Late-Pro leukemias were enriched in distal V_H bins when compared to those in Early-Pro leukemias (Fig. S28c). These data support that the three subtypes are not only arrested at but also arise from different stages of B-cell differentiation.

BCR-ABL1 breakpoint distributions are not different between subtypes and do not affect patient survival

We analyzed the distributions of translocation breakpoints in *BCR* and *ABL1* genes. Fifty-two rearrangements from this study were supplemented with 78 from Score *et al.*[98] and 9 from the

EGAD00001000163 dataset (https://ega-archive.org/datasets). As expected, *BCR* breakpoints were located between exons 13 and 15 in p210 isoform leukemias (Fig. S29a) and between exons 1 and 2 in p190 isoform leukemias (Fig. S29b). All *ABL1* breakpoints were located upstream of exon 2, with a subset even found upstream of the transcription start site in exon 1 (Fig. S29c). Distribution of breakpoints in both genes were not influenced by disease types or molecular subtypes (Figs. S29, S30). In four cases, *BCR-ABL1* translocations were associated with complex rearrangements involving other chromosomes, and in another four cases, resulted in copy number losses of flanking genomic regions (Supplementary Table 15). Gain of Philadelphia chromosome (i.e. +der(22)t(9;22)), found in 9 diagnostic samples (n=9/53, 17%), was not associated with a specific molecular subtype (p=0.38, Fisher's exact test). Furthermore, patient survival was not influenced by *BCR-ABL1* isoforms or gain of Philadelphia chromosome (Fig. S31). These data further support that the molecular subtypes are independent of *BCR-ABL1* rearrangements.

Further evidences of hijacked RAG activity

H3K4me3 histone modification is the binding substrate for RAG2[(99)]. SVs related to cooperating events with RSS motifs were significantly closer to H3K4me3 peaks (transformation SVs with RSS vs. without RSS: 1 kb vs. 18.8 kb; p<1e-16, Wilcoxon rank-sum test; Fig. S32a) and enriched for promoter and enhancer chromatin states (Fig. S32b)[91]. A similar enrichment pattern was previously observed in *ETV6-RUNX1* lymphoblastic leukemia[29]. SVs linked to the *BCR-ABL1* translocation did not harbor this pattern, which further supports that the translocation is not RAG-mediated. Cooperating event SVs with RSS motifs largely consisted of deletions (n=384/399, 96.2%; Fig. S32c) and were significantly smaller in size than cooperating event SVs without RSS motif (71.5 kb vs. 309.5 kb; p=4.1e-6, Wilcoxon rank-sum test; Fig. S32d).

We detected one RAG-mediated rearrangement that was particularly unique and informative. A deletion of *SLX4IP* in Ph18-D had breakpoints at similar locations as other *SLX4IP* deletions, but at the junction, a 75 base-pair 'shard' from the signal sequence of *IGKV4-1* was inserted with non-template sequences at both ends (Fig. S33a). Because such an event is extremely unlikely to occur by chance, we infer that this *SLX4IP* deletion occurred in a spatial and temporal proximity to the *IGKV4-1* recombination before the excised signal sequence, from which the shard is derived, was degraded (Fig. S33b). This may be an example of a recently proposed 'cut-and-run' reaction, in which RAG forms a complex with an excised signal circle to instigate DNA breaks in the genome[100].

A genomic locus upstream of *CBWD2* was recurrently deleted in 36% (n=19/53) of patients and the leftside breakpoints of the deletions were tightly clustered (Fig. S15d). Investigating the DNA sequence near the leftside breakpoint cluster revealed a non-functional, orphan immunoglobulin gene *IGKV1OR2-108* (ENSG00000231292) that provided a canonical RSS motif. Rightside breakpoints were more varied but were positioned near H3K4me3 peaks in the promoter region of *CBWD2*. Six other SVs in our dataset also had breakpoints located near the RSS of 5 different orphan Ig genes: *IGKV3OR2-268* (n=2), *IGKV2OR2-1* (n=1), *IGKV1OR-2* (n=1), *IGHV1OR15-2* (n=1), and *IGHV3OR16-9* (n=1). It can be postulated that these SVs were generated by hijacking the RSS motifs of these orphan Ig genes. Together, these are strong evidences that SVs with cryptic RSS motifs are indeed generated by RAG-mediated recombination.

High number of RAG-mediated recombination is associated with *SLX4IP* deletion

We observed that the total numbers of RAG-mediated recombinations (#RAG) in individual leukemia genomes formed a bimodal distribution (Fig. S34a). Amongst all the genetic and clinical markers, deletion status of *SLX4IP* was most strongly associated with #RAG (FDR-adjusted p=3.7e-5, Wilcoxon rank-sum test; Figs. S34b, 3g). A Poisson regression model using the top nine markers resulted in the best predictor of #RAG, and *SLX4IP* status accounted for the most reduction in residual deviance (Fig. S34c,d). We observed the association between *SLX4IP* deletion and #RAG in 9 *BCR-ABL1* ALL and 40 Ph-like ALL genomes from the European Genome-phenome Archive (EGAD00001000163 and EGAD00001000976)[101]. There was no association between molecular subtypes and #RAG when considering either all cases or only *SLX4IP*-wildtype cases (p=0.10 and p=0.43, Kruskal-Wallis test; Fig. S34e,f).

The cause-and-effect relationship between *SLX4IP* deletion and #RAG is currently uncertain. SLX4IP protein was originally characterized by its interaction with SLX4, a scaffolding protein required for the activity of multiple DNA repair mechanisms, including Holliday junction resolution[102]. Recently, SLX4IP was shown to also regulate a telomere maintenance mechanism known as alternative lengthening of telomeres[103]. Because canonical RAG-mediated recombination is repaired via the non-homologous end joining pathway[104], how the loss of SLX4IP may increase #RAG is not clear. Our study identifies a genetic defect that is associated with elevated RAG-mediated recombination in a lymphoid malignancy.

Blast contamination is common in flow-sorted cell populations

Our findings suggested that Early-, Inter-, and Late-Pro leukemias are transformed at different stages of B-cell development. However, it is possible that they share a common cell-of-origin in which the initiating lesion, *BCR-ABL1*, arises. To tackle this question, we collected stem/progenitor cell populations from leukemia samples using a FACS scheme previously established in the lab[51]. Genomic DNA from each cell population was whole-genome amplified and used for leukemia- and SV-specific nested PCR. A total of 158 SVs (3~16 per patient), including the *BCR-ABL1* translocation of each patient, were assayed for 22 patients. Surprisingly, most cell populations in the cohort displayed a high degree of blast contamination, which was defined as a detection of more than half of the leukemia-specific SVs in a non-blast cell type (Supplementary Table 16). For instance, in Ph12-D, all 14 SVs tested were detected in HSC, MPP, MLP, CMP, and GMP and 12 of the SVs were also detected in T-cells. Because nested PCR is highly sensitive, even a small number of leukemic blasts contaminating a sorted population could result in a false positive signal. Interestingly, greater proportions of assayed cell types were contaminated in Early-Pro samples compared to Inter-Pro or Late-Pro samples (median 80% vs. 50%; p=0.0043, Wilcoxon rank-sum test). We suspect that this difference arises because Early-Pro blasts often express high CD34 and low CD19, as stem/progenitor populations do, and thus are more prone to contaminate non-leukemic cell populations. In Ph16-D (Inter-Pro) and Ph17-D (Late-Pro), *BCR-ABL1* translocation was the sole abnormality detected in HSC, CMP, GMP (Ph17-D only), MEP, and mature B-cells, suggesting that *BCR-ABL1* was the initiating lesion that affected various hematopoietic lineages. However, blast contamination was suspected in the MLP populations of both samples and the GMP population of

Ph16-D. In summary, although nested PCR is a sensitive and efficient method for detecting SVs, widespread blast contamination can hinder data interpretation and needs to be addressed.

TKI-resistant mutations in *BCR-ABL1* develop more frequently in Early-Pro patients

We further explored the differential sensitivity to TKIs observed amongst three subtypes. The kinase domain mutation status during treatment was available on 23 patients. By layering this information on the residual disease plots (Fig. 5a), we observed a striking prevalence of resistance TKI mutations in Early-Pro patients (75%, n=9/12) compared to Inter-Pro (40%, n=2/5) or Late-Pro patients (33%, n=2/6) (Supplementary Table 17). Although our sample size is small, it helps to explain why Early-Pro patients relapse more frequently and specifically benefit from 2nd/3rd generation TKIs (Fig. 5d).

The above observation was partly unexpected given that TKI selection pressure was present in all patients. Kinase domain mutations have been reported to pre-exist at low frequencies at diagnosis[105] and thus it was possible that Early-Pro patients harbor increased prevalence of pre-existing kinase domain mutant clones prior to treatment. To assess this, we performed targeted deep sequencing on 44 diagnostic patient samples. SimSen-seq is a PCR-based approach that utilizes molecular barcodes to increase sensitivity and correct for PCR errors[81]. This method easily captured a clinically detected TKI-resistant mutation, F317L, in a relapse sample, Ph12-R (see Methods). We detected 14 low-frequency kinase domain mutations in 10 out of 44 samples (0.05~1.16%; Fig. S35). Of the 14 mutations, 2 were silent, 1 was a stop-gain, 9 were non-recurrent missense mutations, and 2 were missense mutations previously reported in CML (p.E355G and p.T277A both in Ph40-D)[106,107]. Among these 10 patients, 3 relapsed later but showed no evidence for the selection of pre-existing mutations. Five out of 44 patients relapsed with TKI-resistant kinase domain mutations after 230~865 days from diagnosis, but these mutations were not detected by SimSen-seq at diagnosis. Thus, within our detection limits, Early-Pro patients do not show increased frequency of pre-existing TKI resistant mutations at diagnosis. This suggests that resistance mechanisms in Early-Pro patients may contribute to a predisposition towards developing kinase domain mutations.

Fig. S1 | Non-negative matrix factorization (NMF) of RNA-seq data

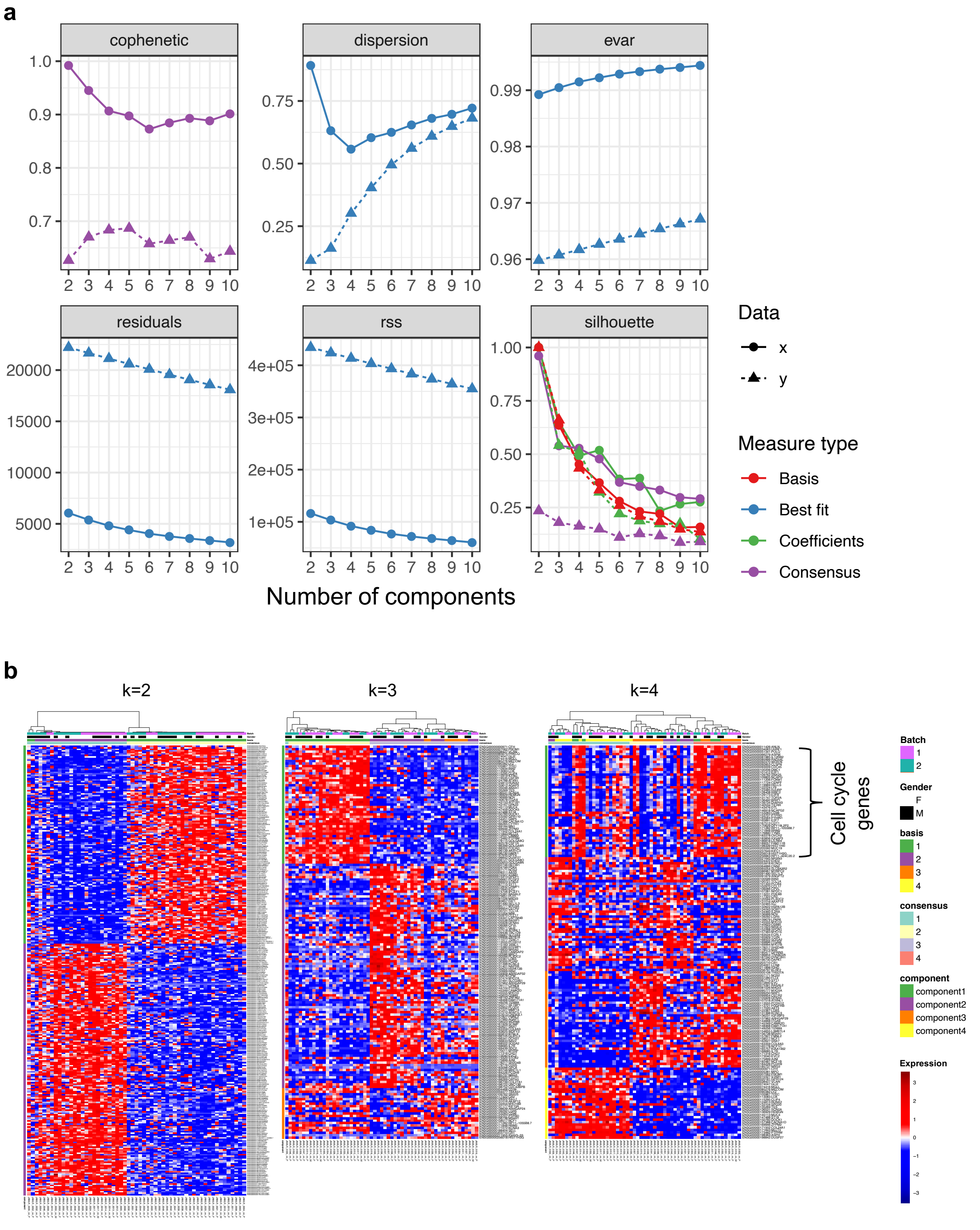

a, Comparison of quality measures from the actual data (solid line) and from randomized data (dashed line) for numbers of components (k) between 2 and 10. **b**, Gene expression heatmaps of NMF component genes for k values between 2 and 4. Rows represent genes and columns represent samples. Genes are grouped into NMF components.

Fig. S2 | Identification of RNA-seq subtypes by consensus hierarchical clustering

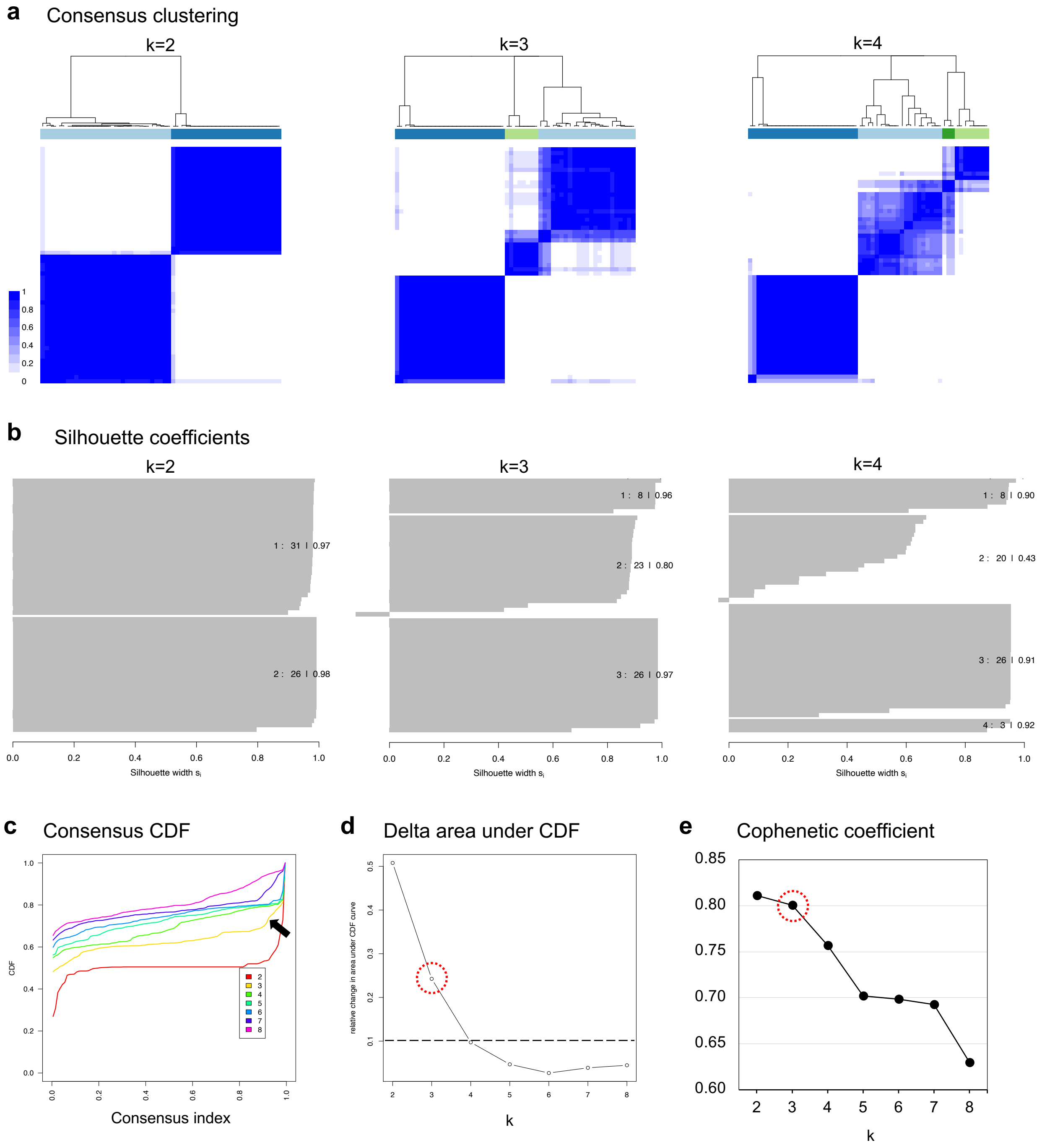

a-e, Consensus hierarchical clustering was performed using 163 genes from 3 NMF components. Quality measures were compared across numbers of clusters (k) to determine the most stable number of clusters. **a**, Consensus clustering matrix for k=2~4. Cell shade represents the proportion of re-samplings where two samples are clustered together. **b**, Silhouette coefficients for k=2~4. **c**, Empirical cumulative distribution function (CDF) plot. **d**, Difference in area under CDF curves of two consecutive k values. **e**, Cophenetic coefficient.

Fig. S3 | Three molecular subtypes in an independent cohort of 40 *BCR-ABL1* ALL cases identified by 3'-seq

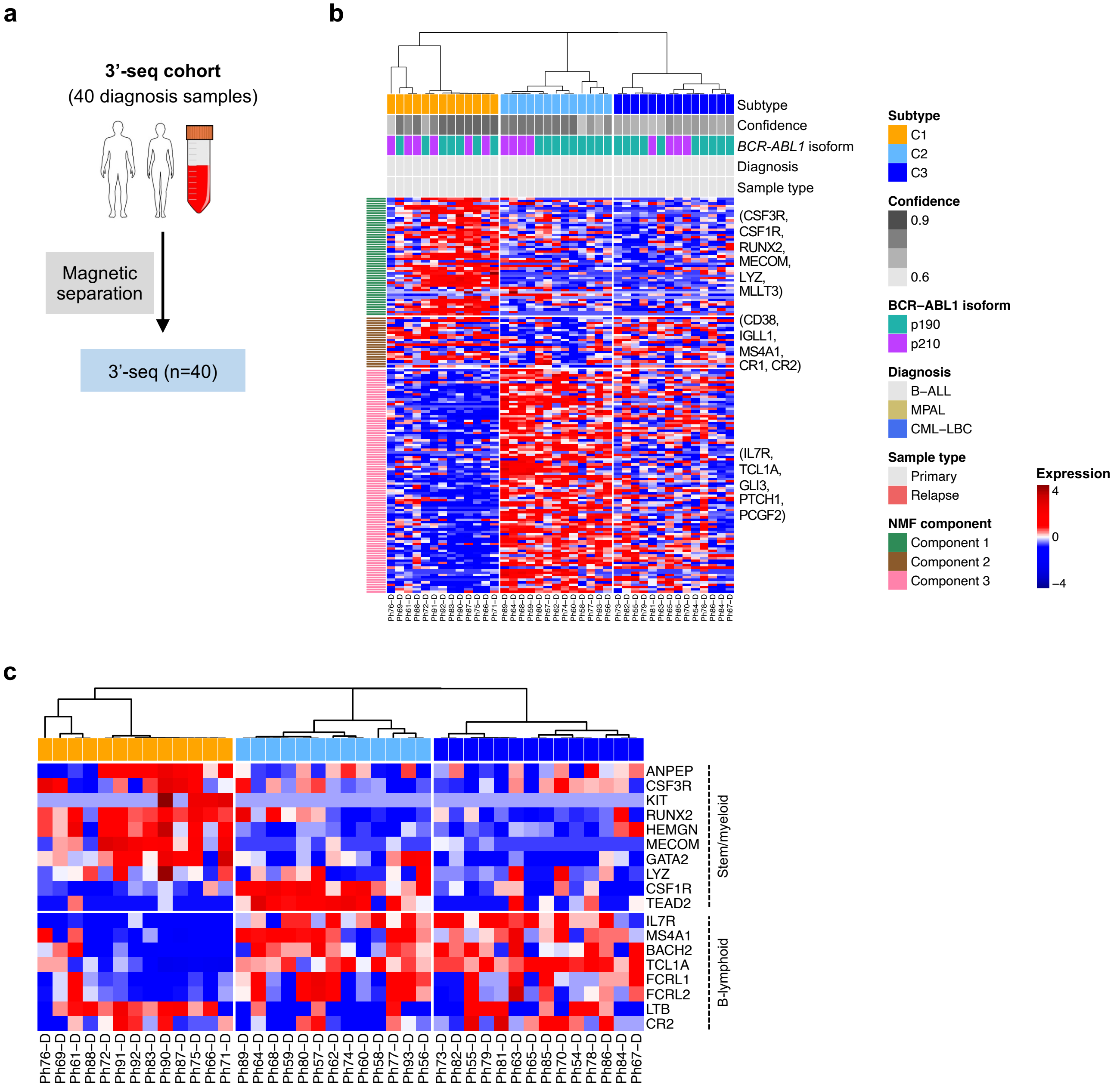

a, Independent cohort of 40 *BCR-ABL1* B-ALL patients. Samples were enriched for leukemic blasts using CD19⁺ magnetic separation and then profiled using 3'-seq. **b**, Gene expression heatmap of three molecular subtypes in the 3'-seq cohort identified by consensus hierarchical clustering. See Fig. 1a for legend. **c**, Heatmap of marker genes in stem/myeloid and B-lymphoid programs. Sample order and heatmap scale are the same as in **b**.

Fig. S4 | Consensus hierarchical clustering of the second cohort

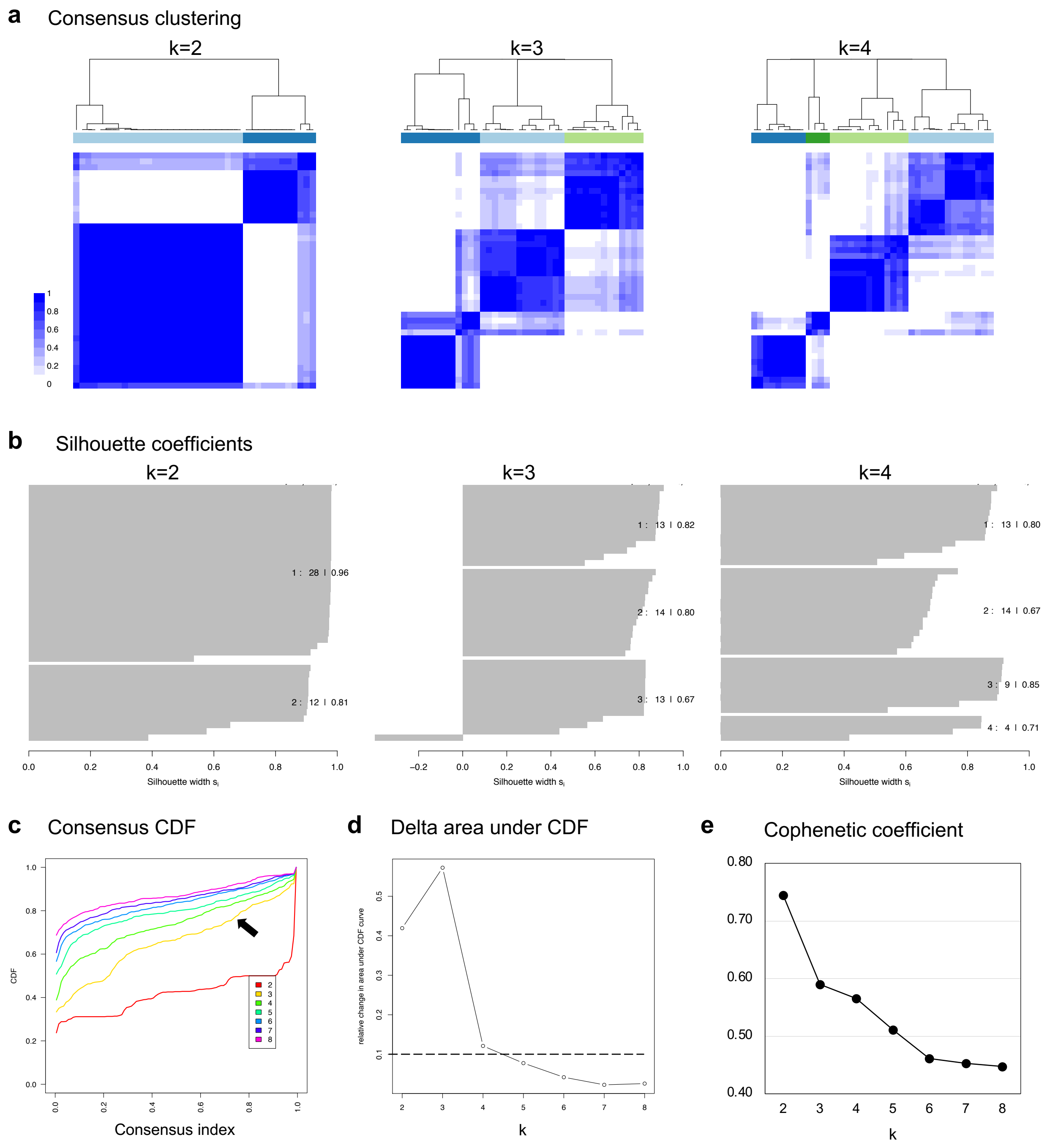

Consensus hierarchical clustering of the second cohort using the original 163 NMF component genes. See Fig. S2 for legend.

Fig. S5 | Clustering of 30 Ph-like ALL

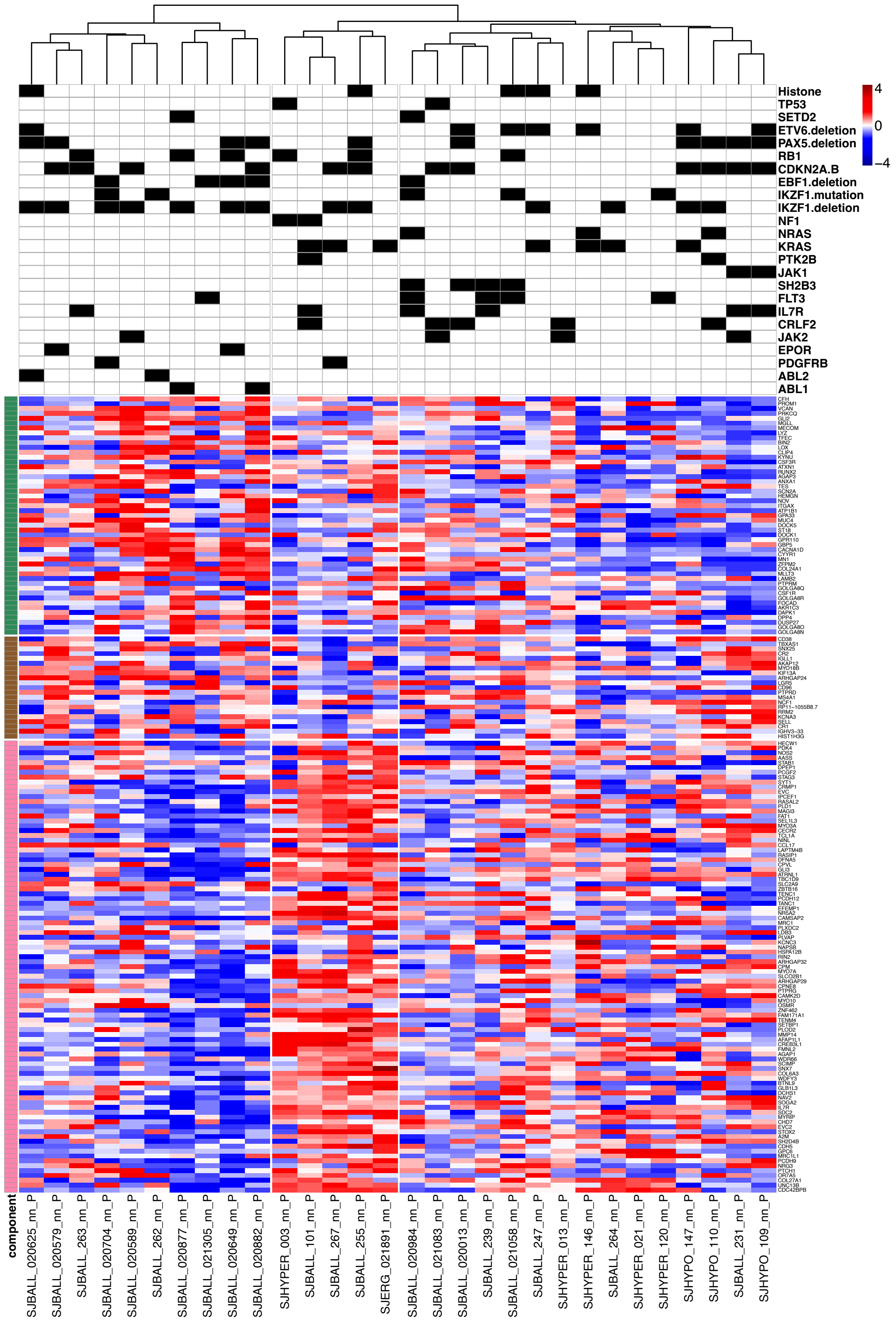

Clustering of 30 Ph-like ALL from EGAD00001001016 dataset[101].

Fig. S6 | Gene set enrichment analysis (GSEA) of each subtype against the rest

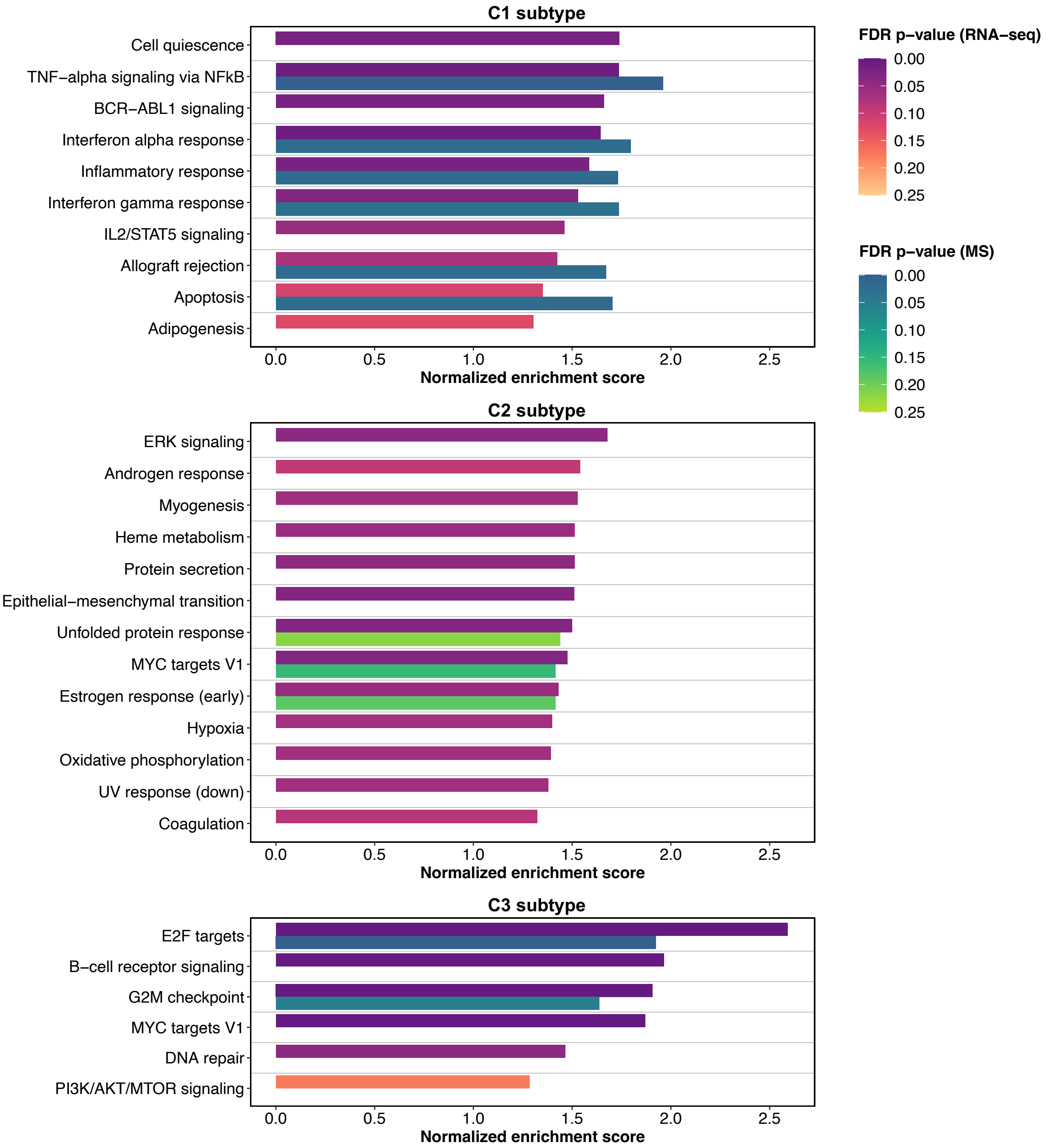

For each subtype, enriched gene sets with nominal p-value<0.05 and FDR-adjusted p-value<0.25 from the RNA-seq data are shown. For each gene set, top bars represent normalized enrichment score (NES) from the RNA-seq data and bottom bars represent NES from mass spectrometry (MS) data. Color of the bar corresponds to the FDR-adjusted p-value.

Fig. S7 | Proportion of lineage marker-positive blasts

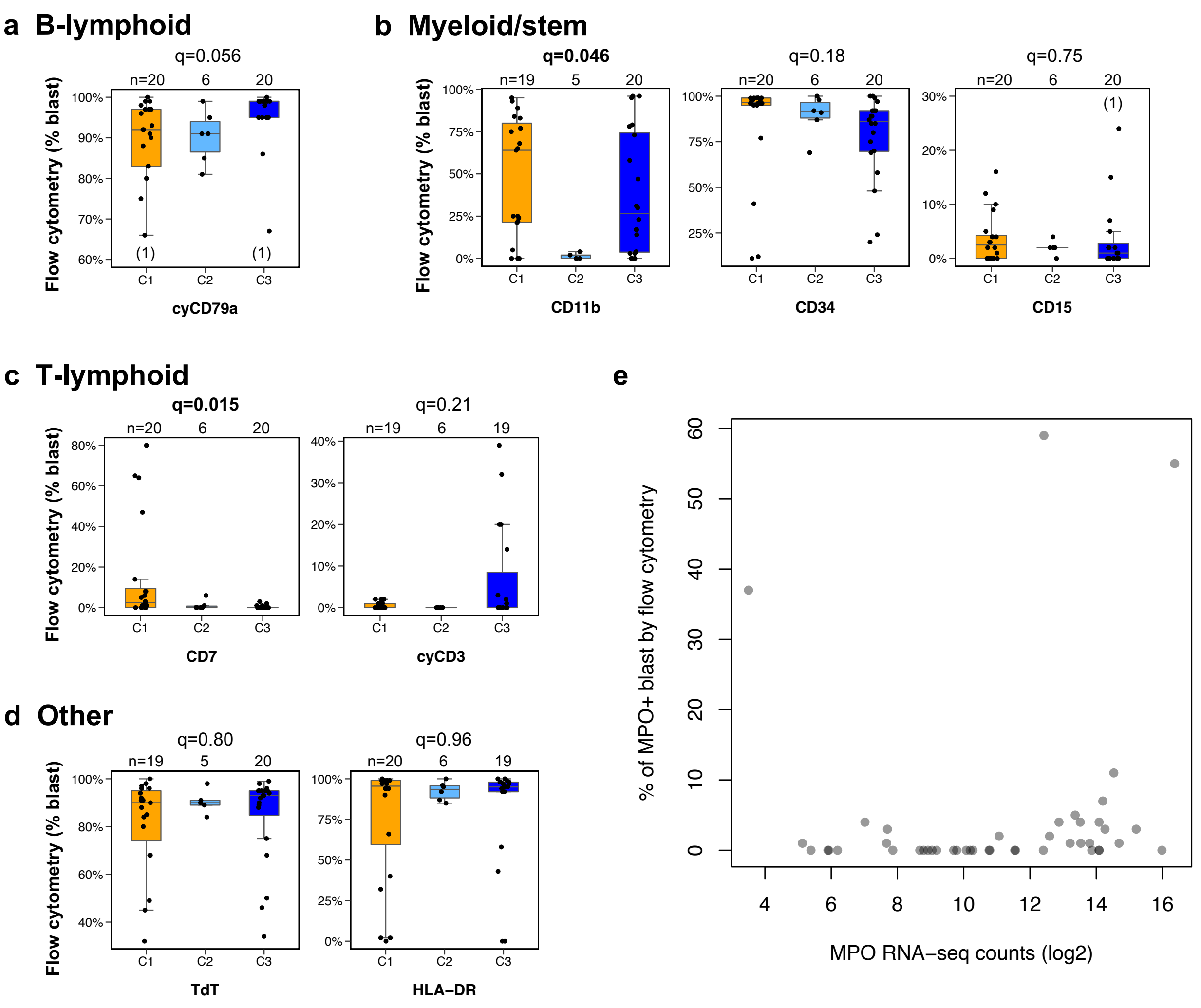

a-d, Proportions of blasts positive for lineage marker antigens by subtype. **a**, B-lymphoid, **b**, myeloid/stem, **c**, T-lymphoid, and **d**, other markers. Numbers in brackets indicate outliers that are not shown to improve visibility of the overall comparison. Counts represent numbers of primary leukemias assessed for the antigen. FDR-adjusted p-values from Kruskal-Wallis test are shown. **e**, Comparison of MPO expression by RNA-seq (log2 counts) vs. flow cytometry *(*% of MPO-positive blasts).

Fig. S8 | Expression of lineage marker genes

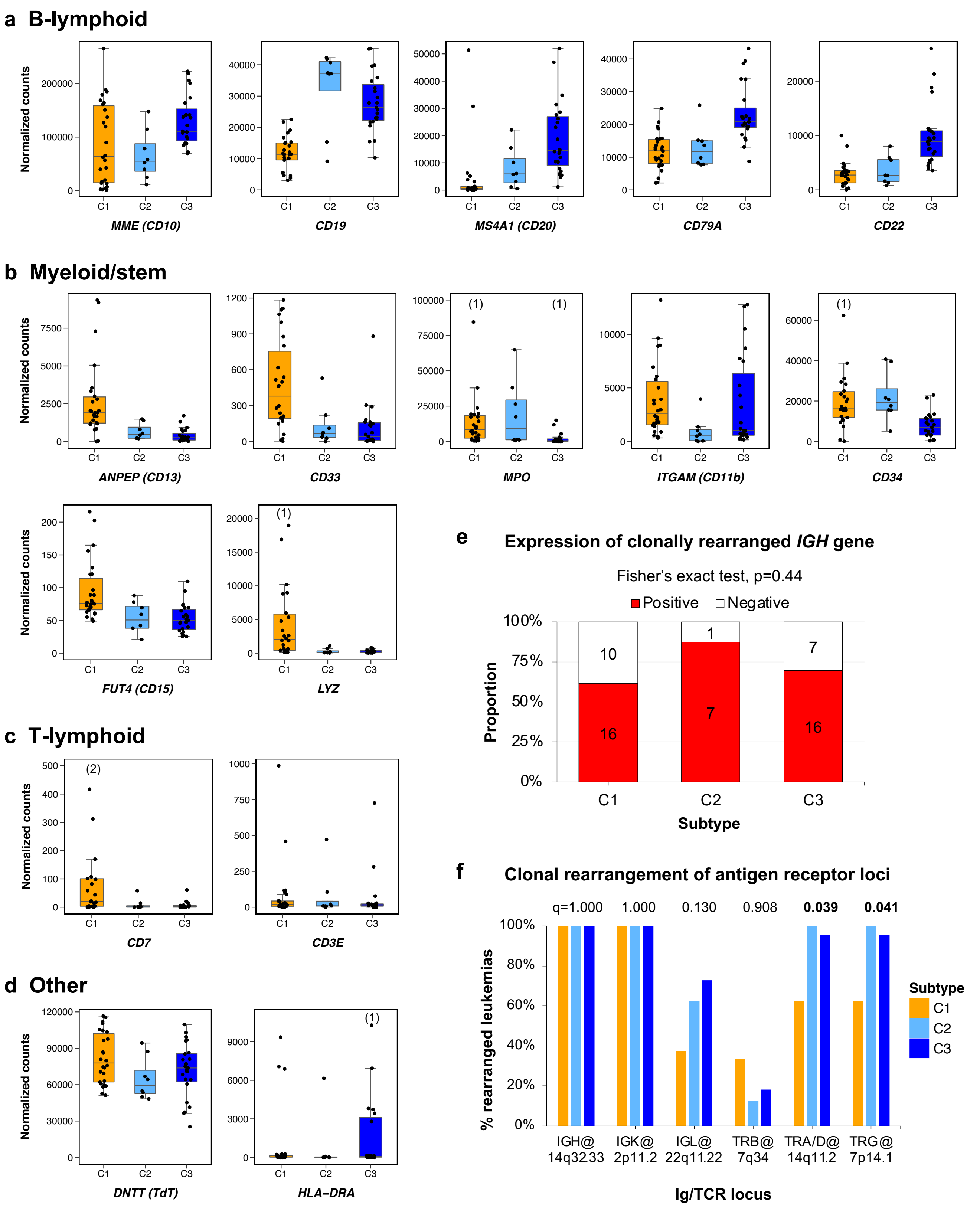

a-d, Normalized counts of lineage marker genes by subtype in the main cohort (26 C1, 8 C2, 23 C3). **a**, B-lymphoid, **b**, myeloid/stem, **c**, T-lymphoid, and **d**, other marker genes. **e**, Proportions of leukemias in each subtype with and without expression of clonally rearranged immunoglobulin heavy chain gene (*IGH*). p-value is from Fisher's exact test. **f,** Frequencies of clonal rearrangements in the immunoglobulin (*IGH*, *IGK*, *IGL*) and T-cell receptor (*TRB*, *TRA/D*, *TRG*) loci by subtype. FDR-adjusted p-values from Fisher's exact test are shown. **g-j**, Normalized counts of lineage marker genes by subtype in the 3'-seq cohort (13 C1, 13 C2, 14 C3). **g**, B-lymphoid, **h**, myeloid/stem, **i**, T-lymphoid, and **j**, other marker genes. Numbers in brackets indicate outliers that are not shown to improve visibility of the overall comparison.

Fig. S8 | Expression of lineage marker genes (continued)

g B-lymphoid

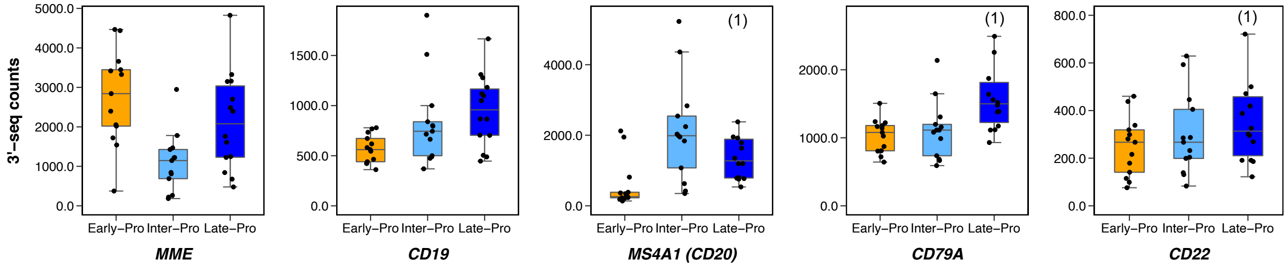

h Myeloid/stem

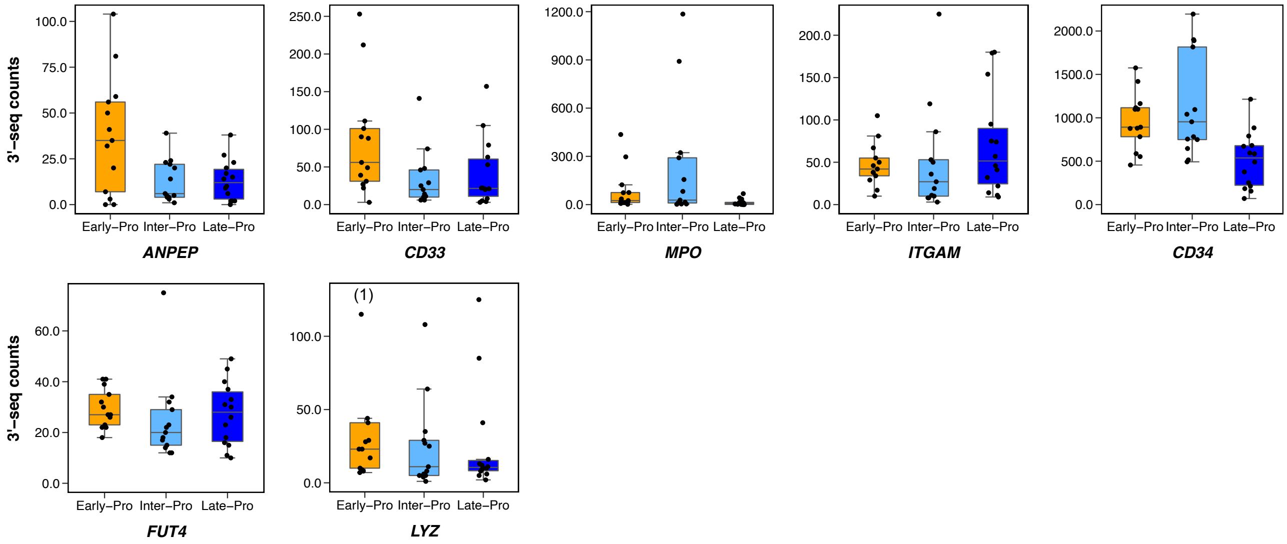

i T-lymphoid

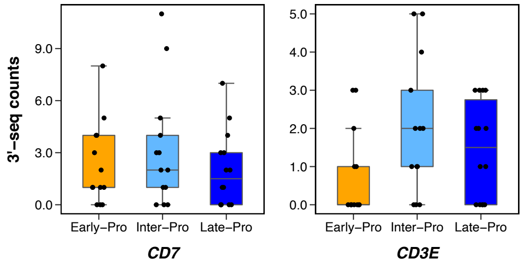

j Other

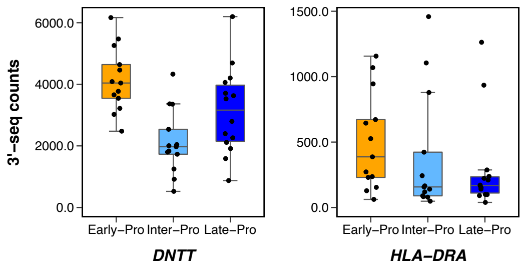

Fig. S9 | Reference dataset of hematopoietic cell compartments from human cord blood

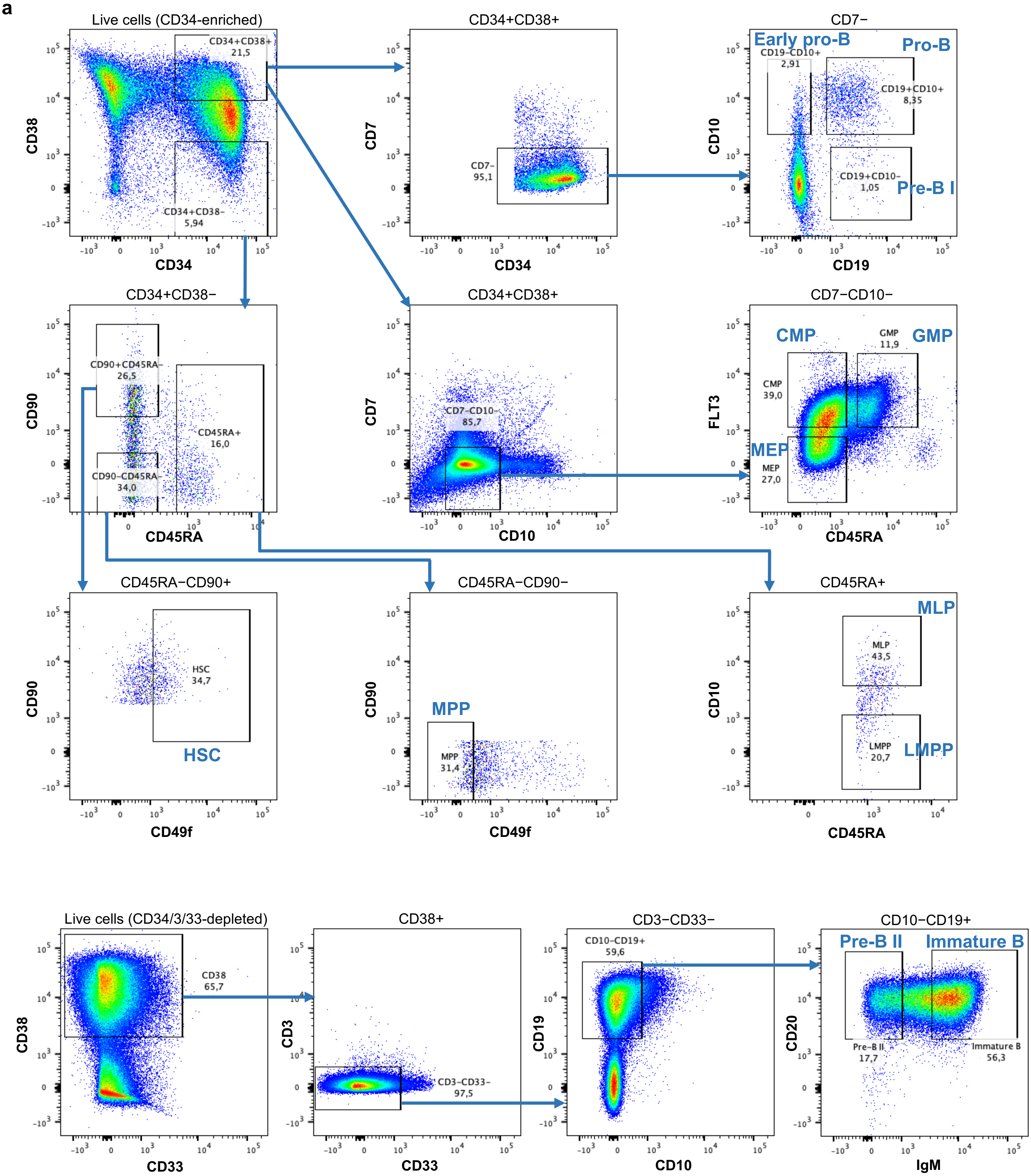

a, Flow cytometry scheme used to isolate hematopoietic stem and progenitor cell compartments from human cord blood. **b,** Gene expression of EBF1, PAX5, DNTT, RAG1, RAG2, and B-cell receptor signaling across leukemia subtypes and normal cord blood cell compartments (counts are shown at the top). **c**, Leukemia samples were scored for gene expression signatures specific to cell populations in adult bone marrow[14]. Mean scores for Early-Pro, Inter-Pro and Late-Pro subtypes are shown.

Fig. S9 | Reference dataset of hematopoietic cell compartments from human cord blood (continued)

b

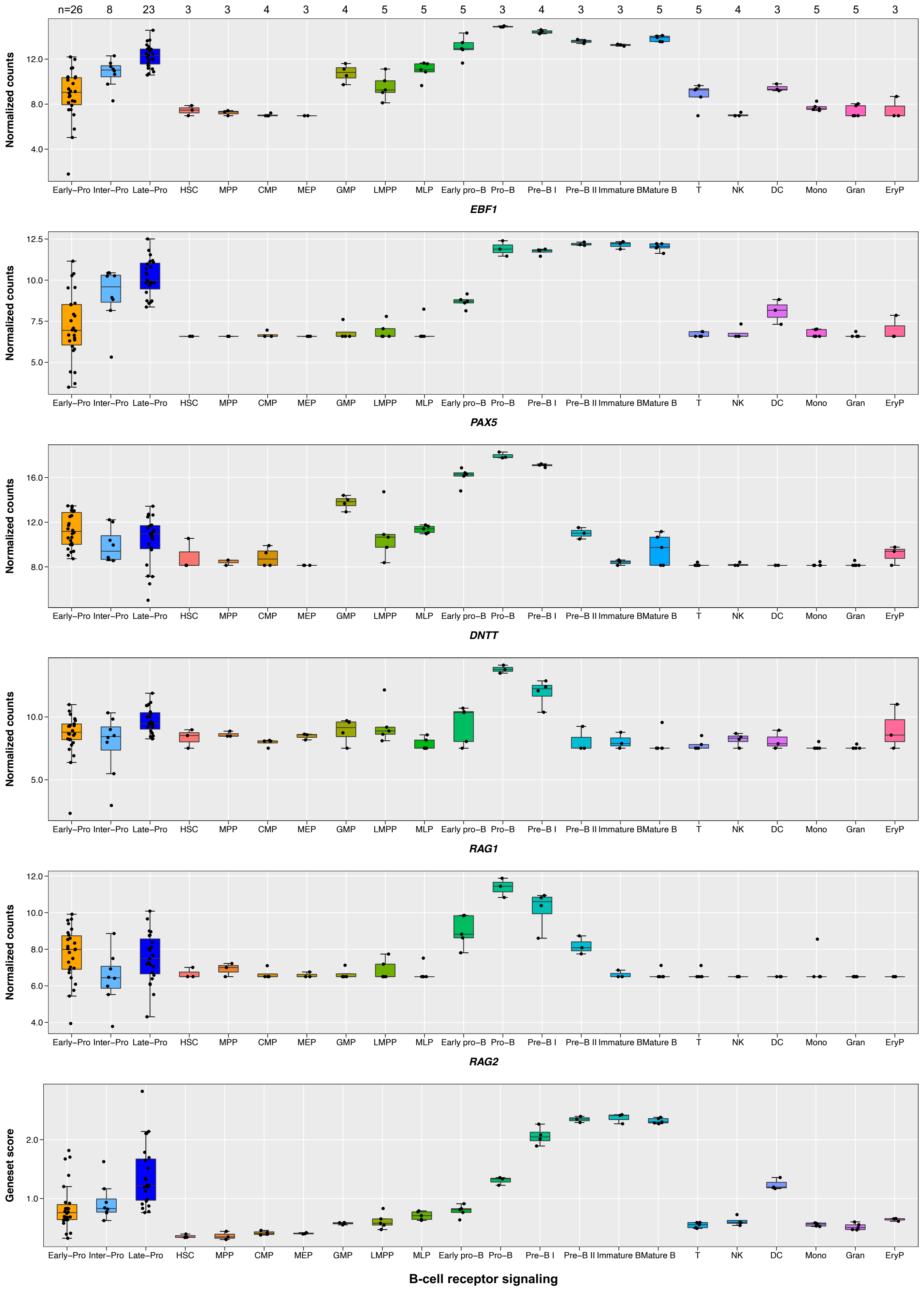

Fig. S9 | Reference dataset of hematopoietic cell compartments from human cord blood (continued)

c

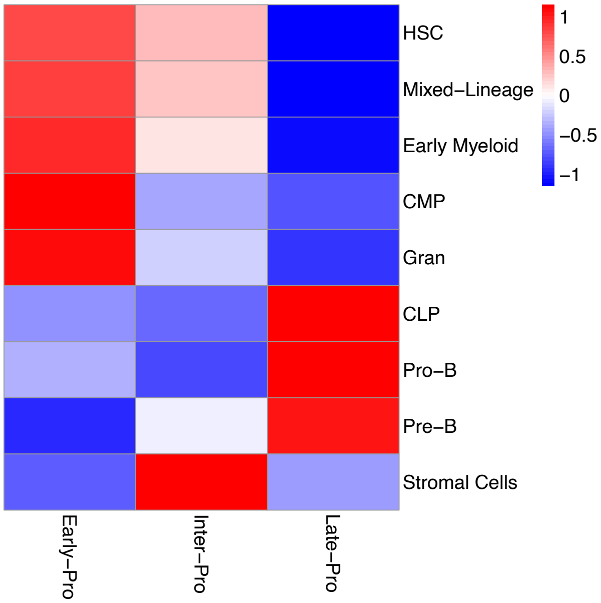

Fig. S10 | Rearrangements of antigen receptor loci

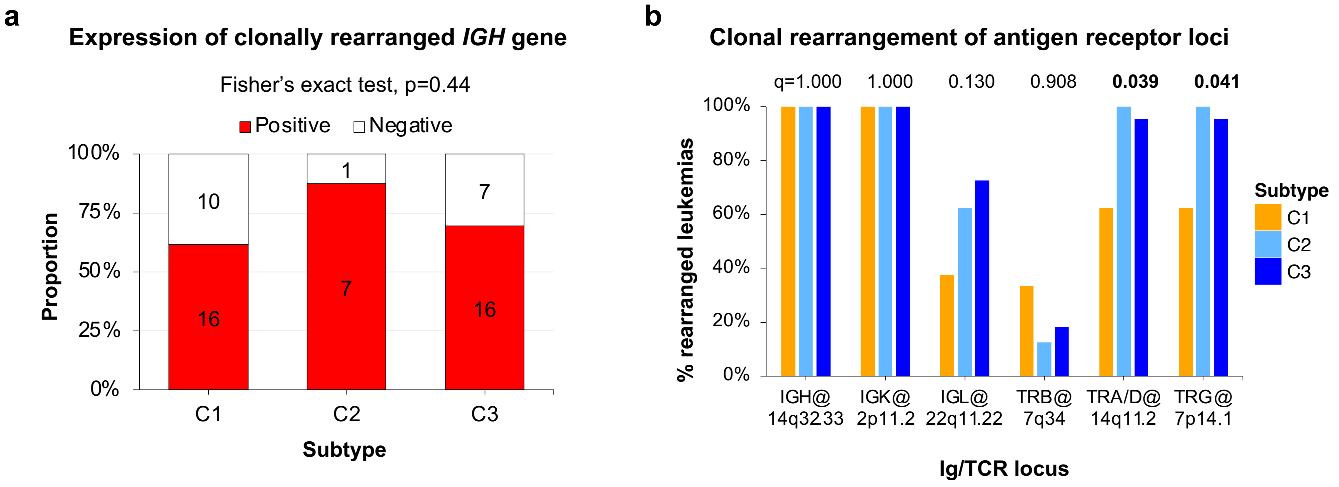

a, Proportions of leukemias in each subtype with and without expression of clonally rearranged immunoglobulin heavy chain gene (*IGH*). p-value is from Fisher's exact test. **b,** Frequencies of clonal rearrangements in the immunoglobulin (*IGH*, *IGK*, *IGL*) and T-cell receptor (*TRB*, *TRA/D*, *TRG*) loci by subtype. FDR-adjusted p-values from Fisher's exact test are shown.

Fig. S11 | Annotation of single-cell RNA-seq samples using adult bone marrow data

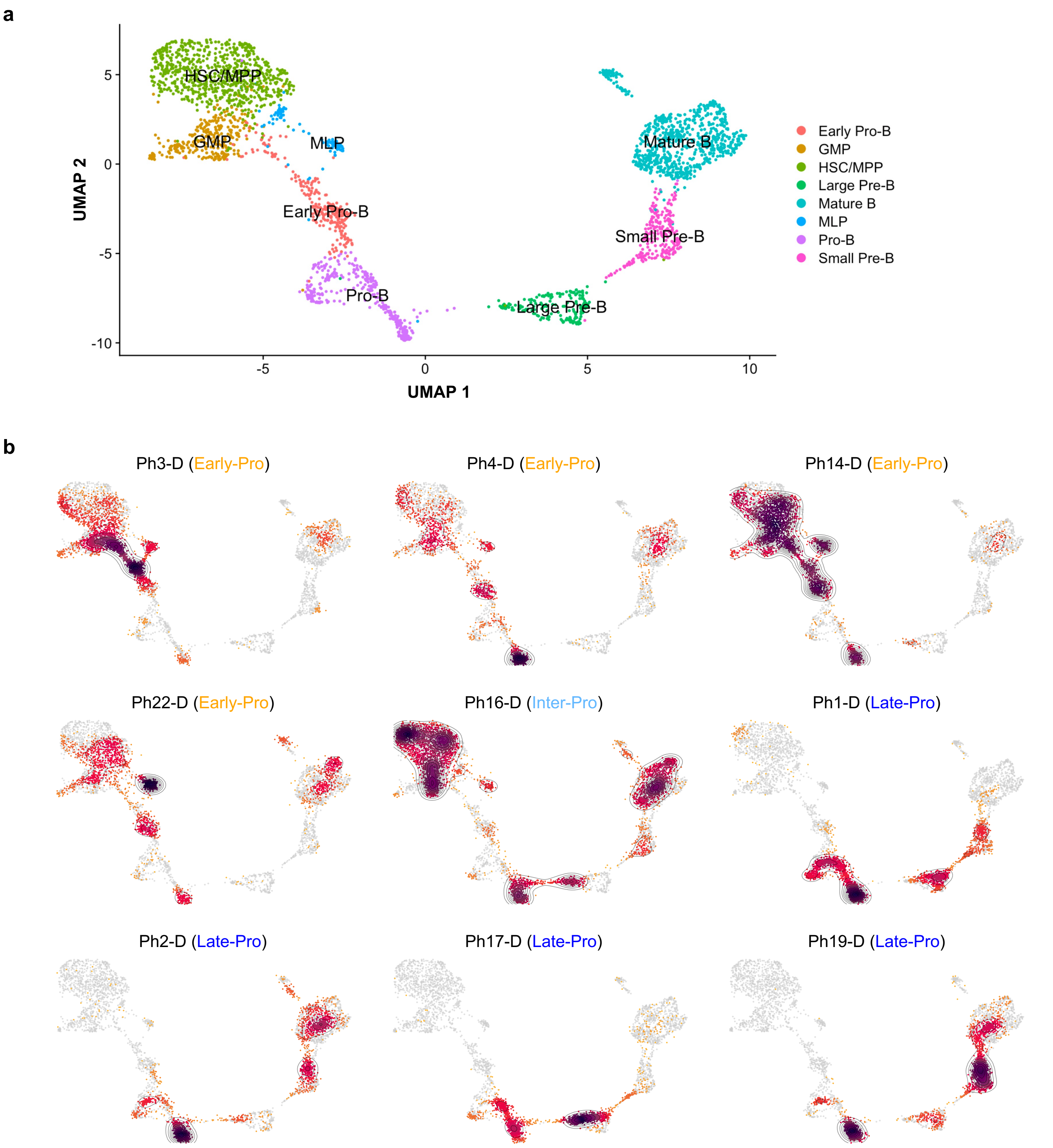

a, UMAP visualization of 3032 cells from an adult bone marrow spanning HSC to mature B utilizing scRNA-seq and Abseq data from Triana *et al.*[19]. Single cell clusters are labelled based on prior annotations, RNA marker genes, and protein-level surface markers. **b**, Nine scRNA-seq samples are projected onto the B-cell development trajectory of an adult bone marrow using Symphony[71]. Red dots indicate leukemic cells. Each leukemic cell is assigned a cell type label based on 30 nearest-neighbours within the reference dataset. **c**, Mean proportions of cell type labels for Early-Pro, Inter-Pro and Late-Pro subtypes. **d**, Pseudotime analysis of 4 Early-Pro scRNA-seq samples using Monocle 3[(68)]. Trees and their branches show the trajectory of single cells and their cell type annotations. Barplots at the bottom show cell type annotation counts across pseudotime (left to right).

Fig. S11 | Annotation of single-cell RNA-seq samples using adult bone marrow data (continued)

c

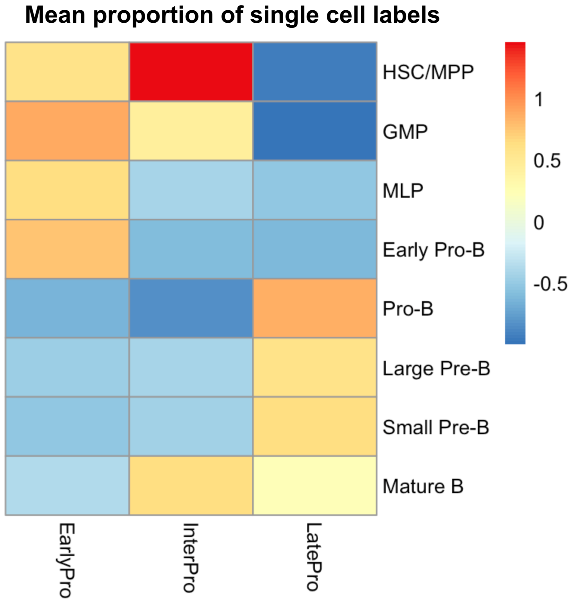

d

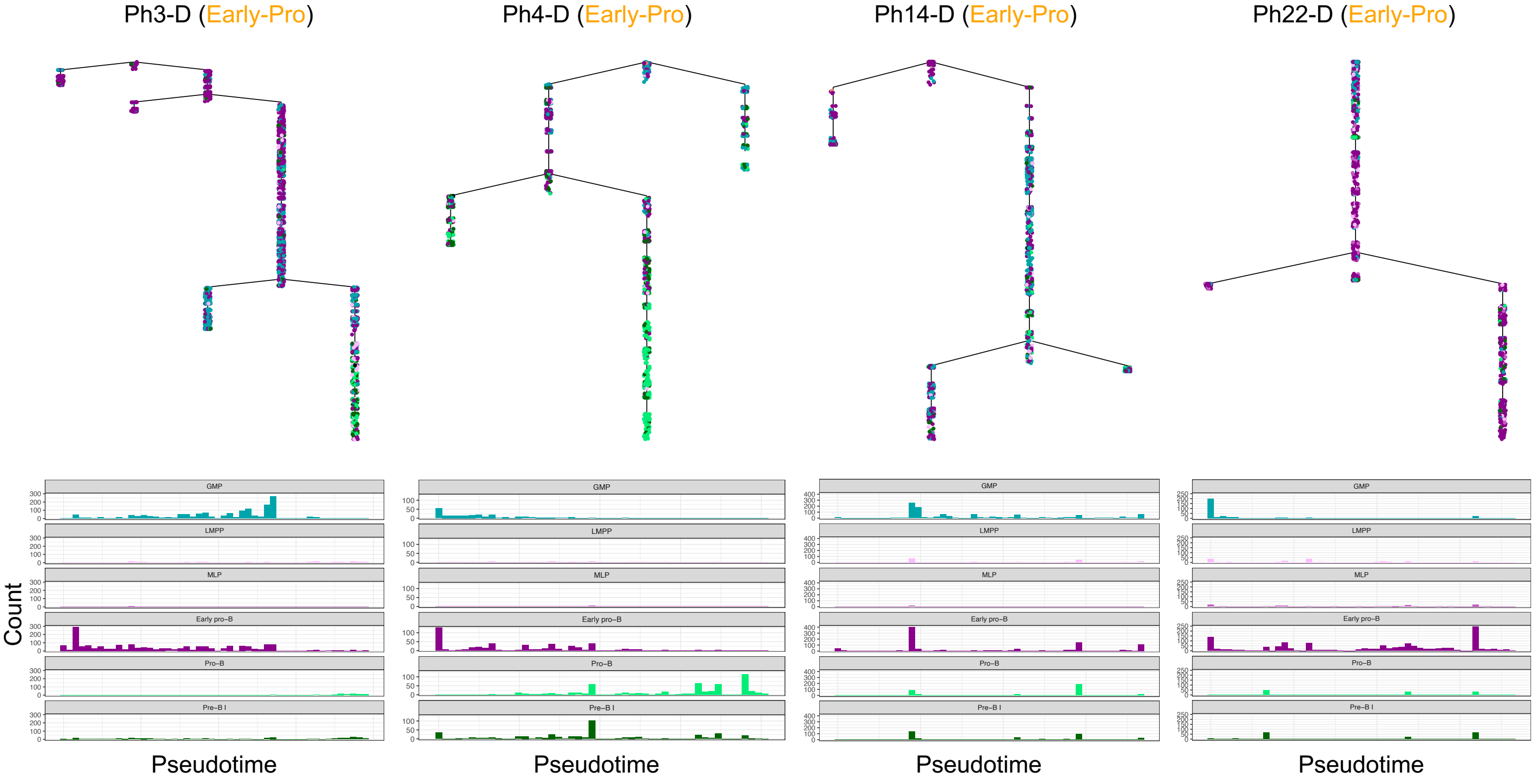

Fig. S12 | Leukemias with intermediate gene expression profiles

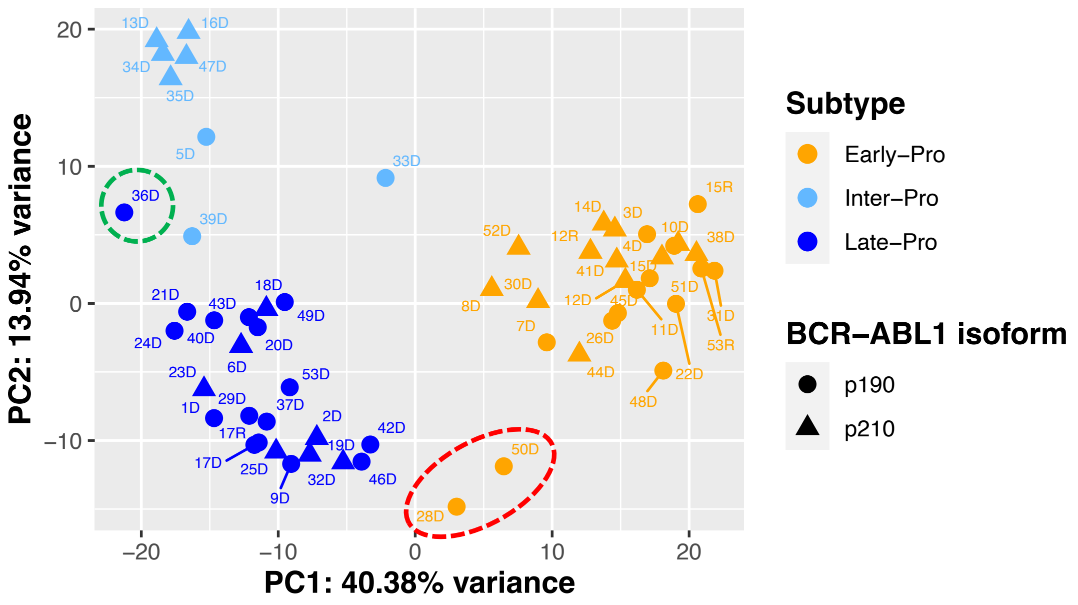

PCA visualization of *BCR-ABL1* lymphoblastic leukemia transcriptomes using 163 NMF component genes. Red circle highlights the positions of Ph28-D and Ph50-D, which are classified as Early-Pro and positioned between Early-Pro and Late-Pro subtypes. They both harbor concurrent losses of genes associated with Early-Pro subtype (*RUNX1* and *EBF1*) as well as with Late-Pro subtype (*PAX5* and *CDKN2A/B*). Green circle highlights Ph36-D, which is classified as Late-Pro and positioned close to Inter-Pro subtype. It harbors losses of *PAX5* and *RB1* and a dominant-negative *IKZF1* mutation (p.N159T)[24].

Fig. S13 | Frequency of large-scale copy number alterations in diagnostic leukemias

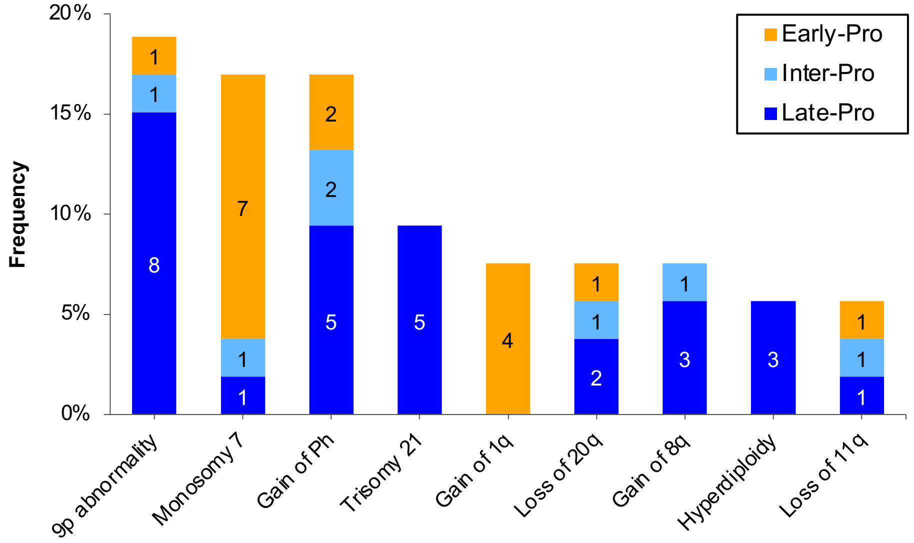

Frequency of large-scale copy number alterations (i.e. chromosome- or arm-level events) in diagnostic leukemias (n=53). For each alteration, the numbers of leukemias in three subtypes are shown.

Fig. S14 | Recurrent large-scale copy number alterations

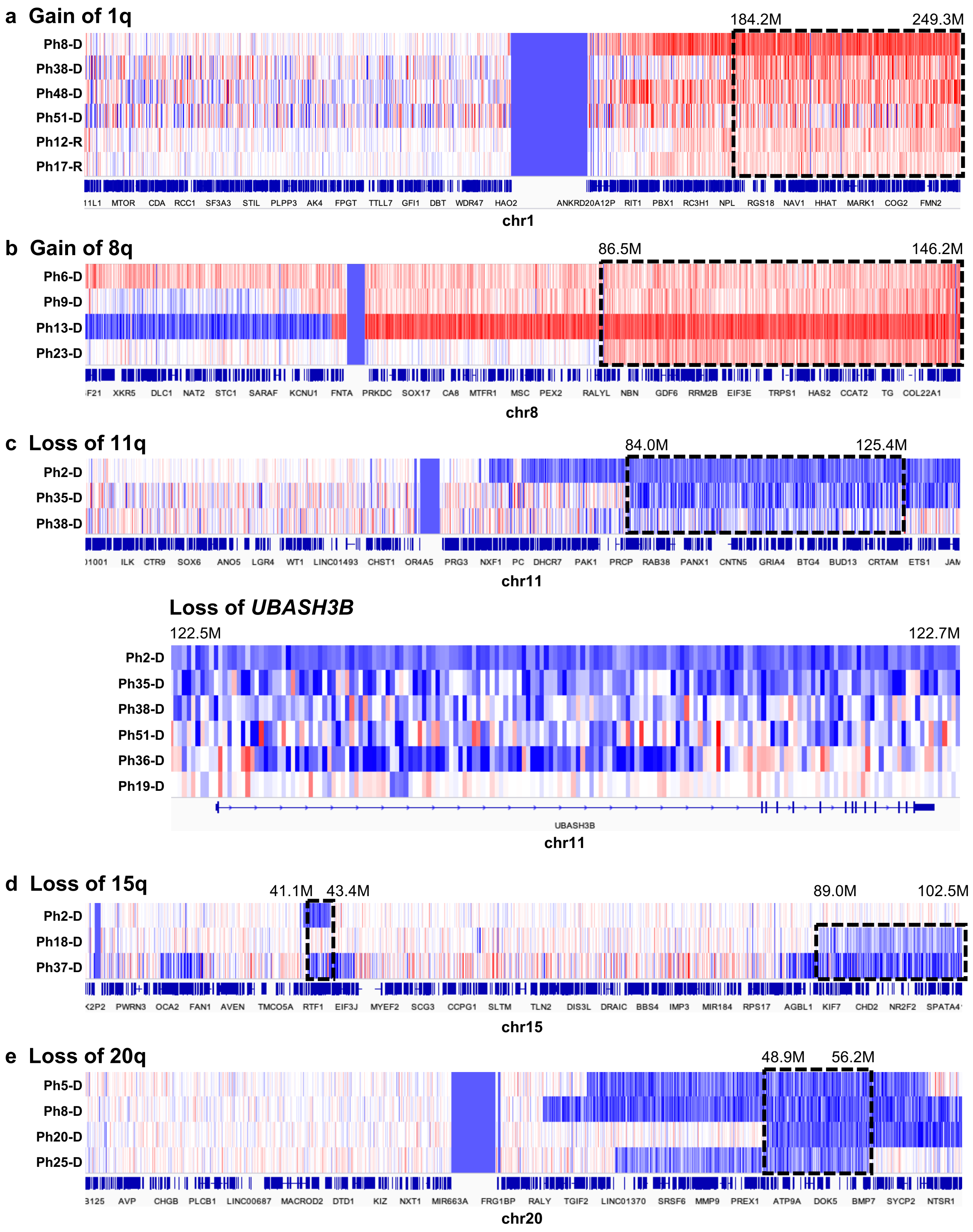

a-e, Recurrent large-scale copy number alterations visualized using the Integrative Genomics Viewer (IGV). Blue represents copy number loss and red represents copy number gain relative to the reference T-cell genome. Dashed boxes with chromosome positions denote minimal common regions. **a**, Gain of 1q. **b**, Gain of 8q. **c**, Loss of 11q (lower panel displays smaller copy number losses encompassing *UBASH3B* in 3 additional cases). **d**, Loss of 15q. **e**, Loss of 20q.

Fig. S15 | RAG-mediated recombination generates recurrent secondary alterations

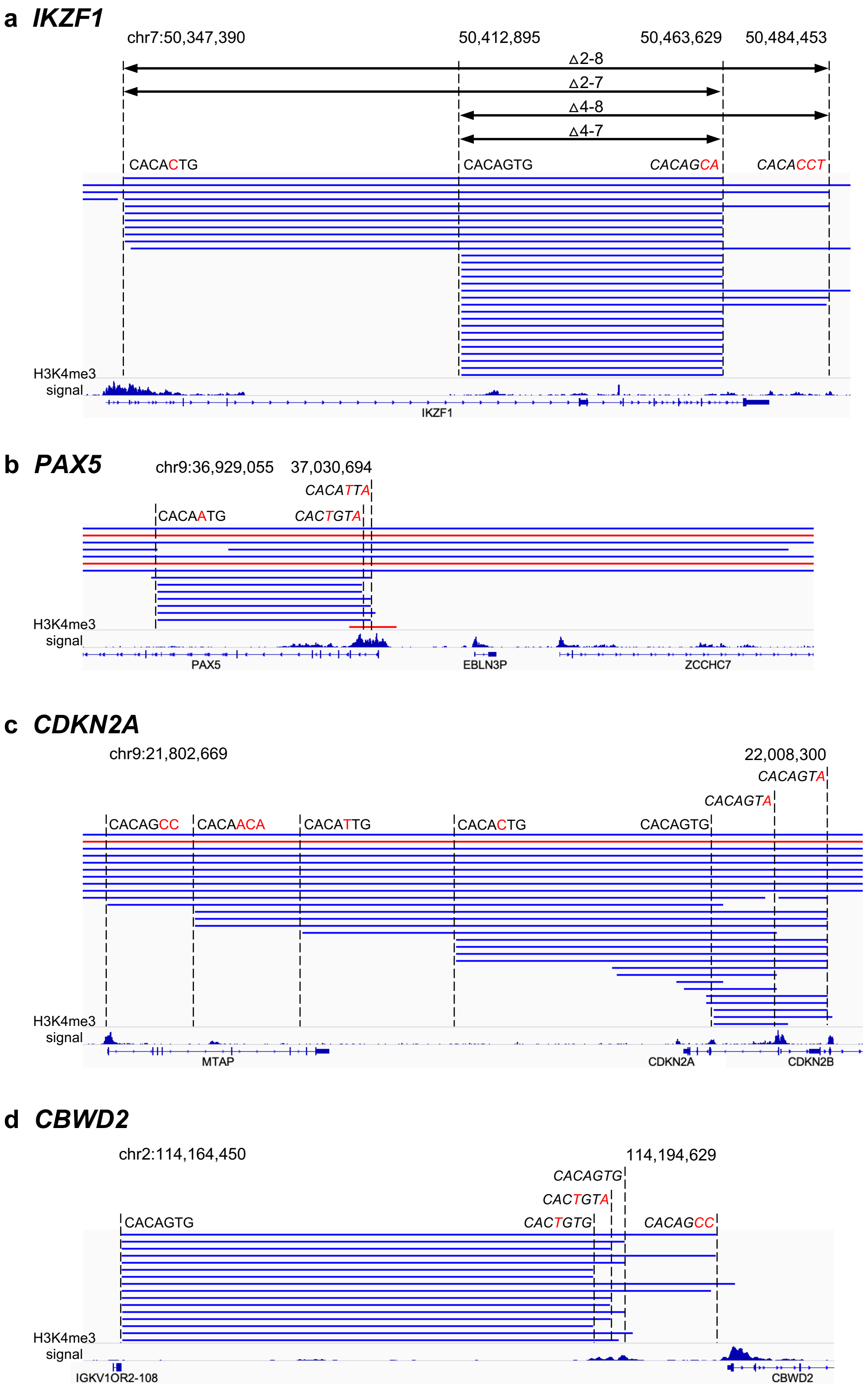

Generation of recurrent secondary alterations by RAG-mediated recombination. Deletions (blue lines) and insertions (red lines) are visualized using the Integrative Genomics Viewer (IGV). Exemplar RSS motifs are displayed inside breakpoint clusters, which are marked with dashed lines. Bases that deviate from the canonical RSS motif (CACAGTG) are in red font. Rightside RSS sequences are written in reverse complement and italicized. H3K4me3 ChIP-seq signals for GM12878 (B-lymphoblast cell line) from ENCODE are shown at the bottom.

Fig. S15 | RAG-mediated recombination generates recurrent secondary alterations (continued)

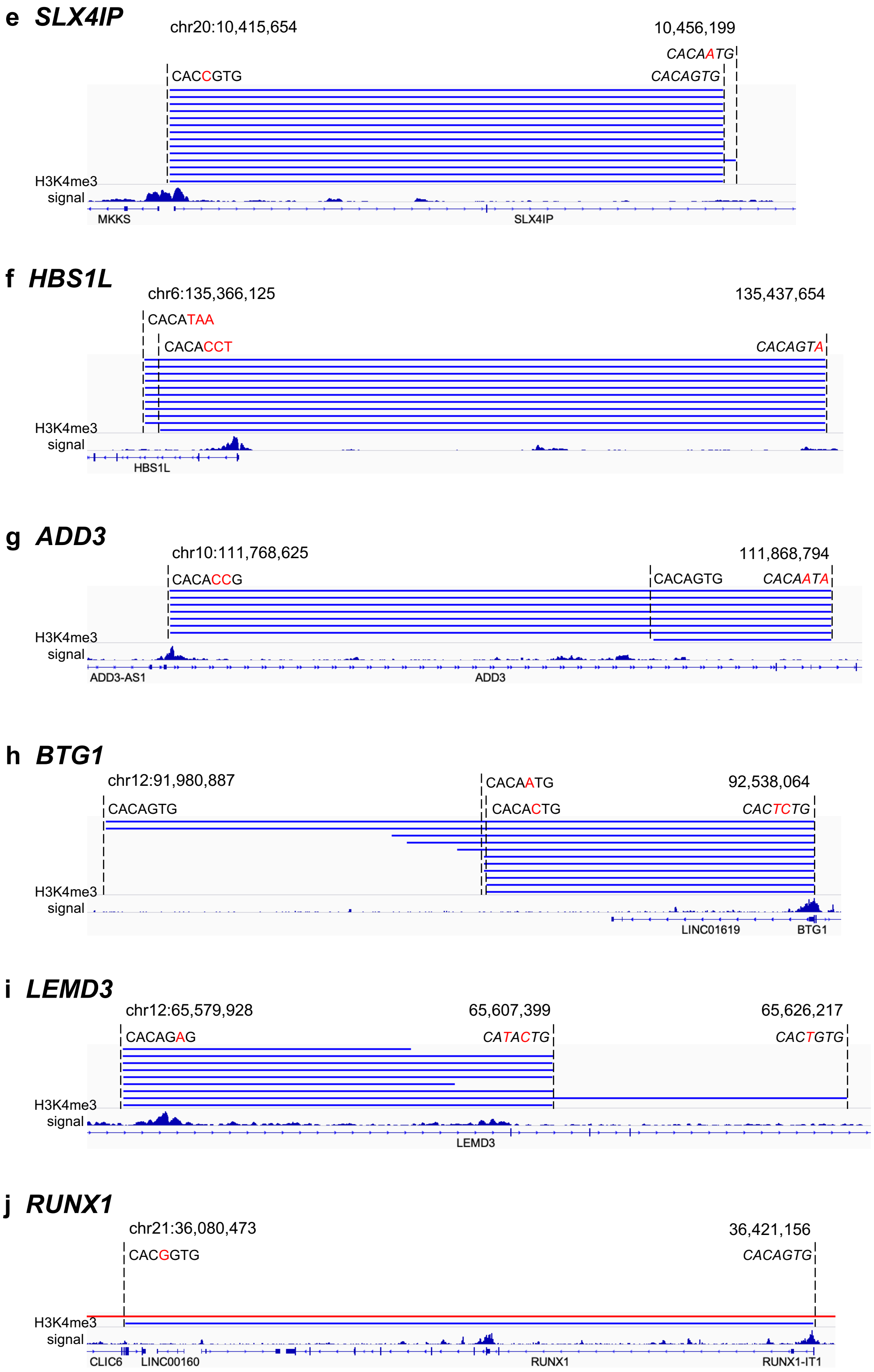

Fig. S15 | RAG-mediated recombination generates recurrent secondary alterations (continued)

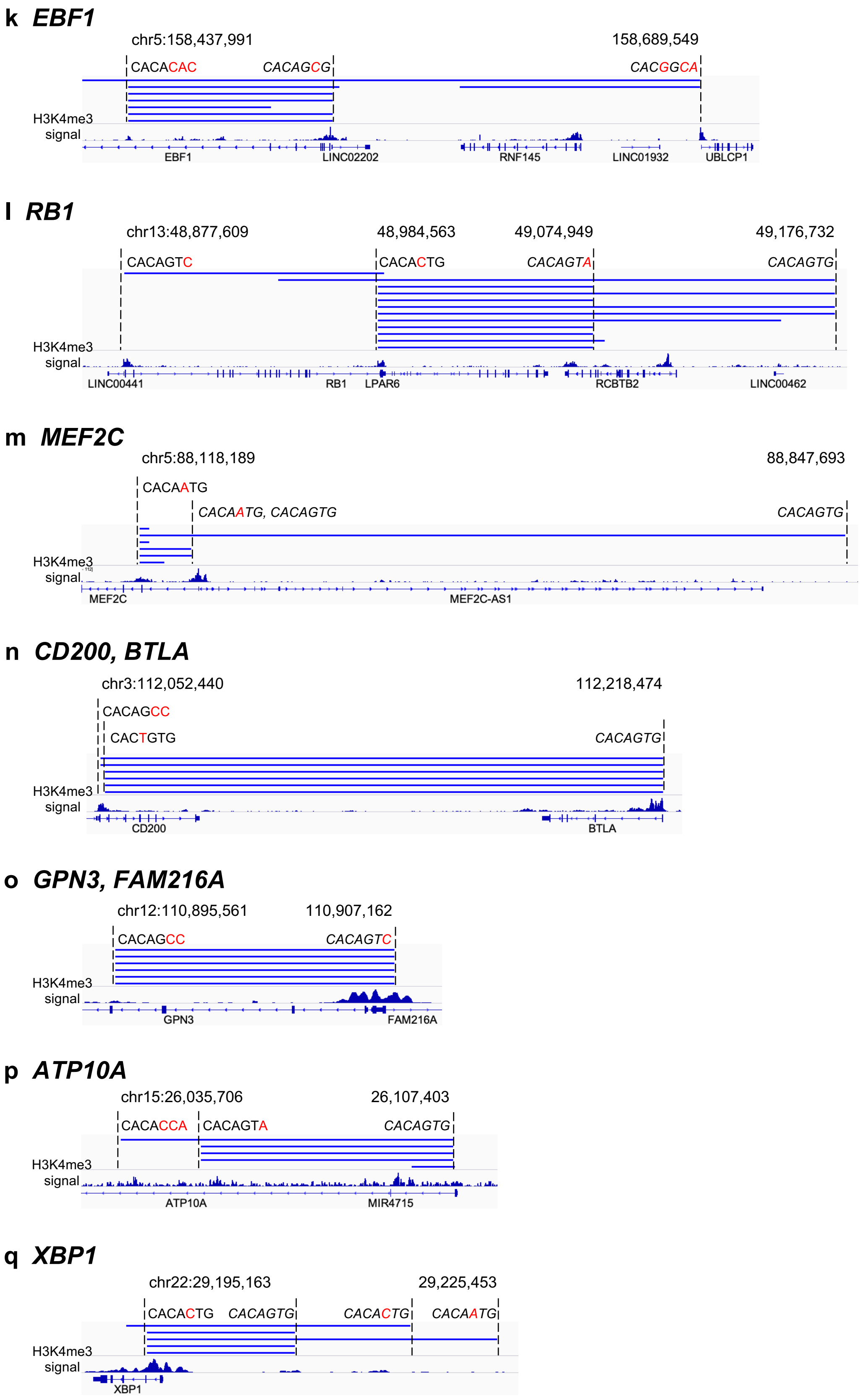

Fig. S15 | RAG-mediated recombination generates recurrent secondary alterations (continued)

r *PRKAR2B*

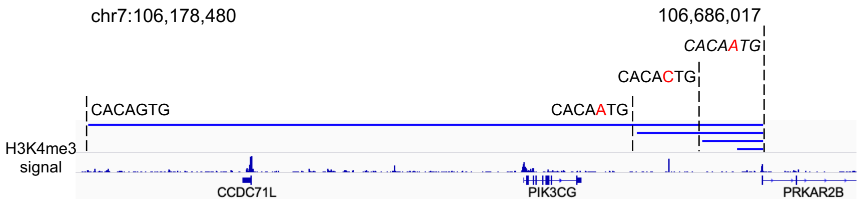

s *SMIM3*

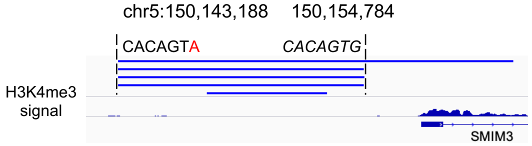

t *GAB1*

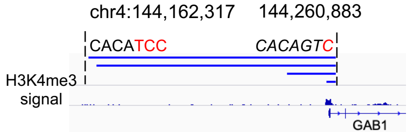

u *TSC22D1, SERP2*

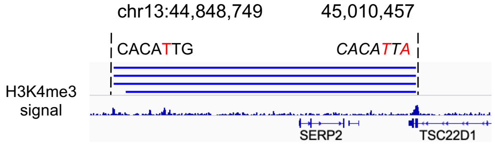

v *RAG2*

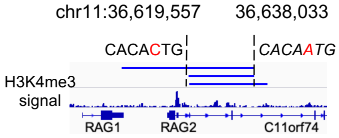

w *MAP3K2*

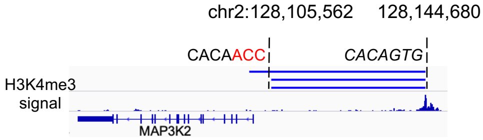

x *MIR181A1HG*

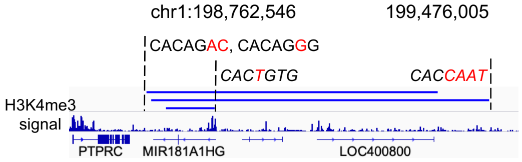

Fig. S16 | Comparison of NTS insertion and microhomology across SV categories

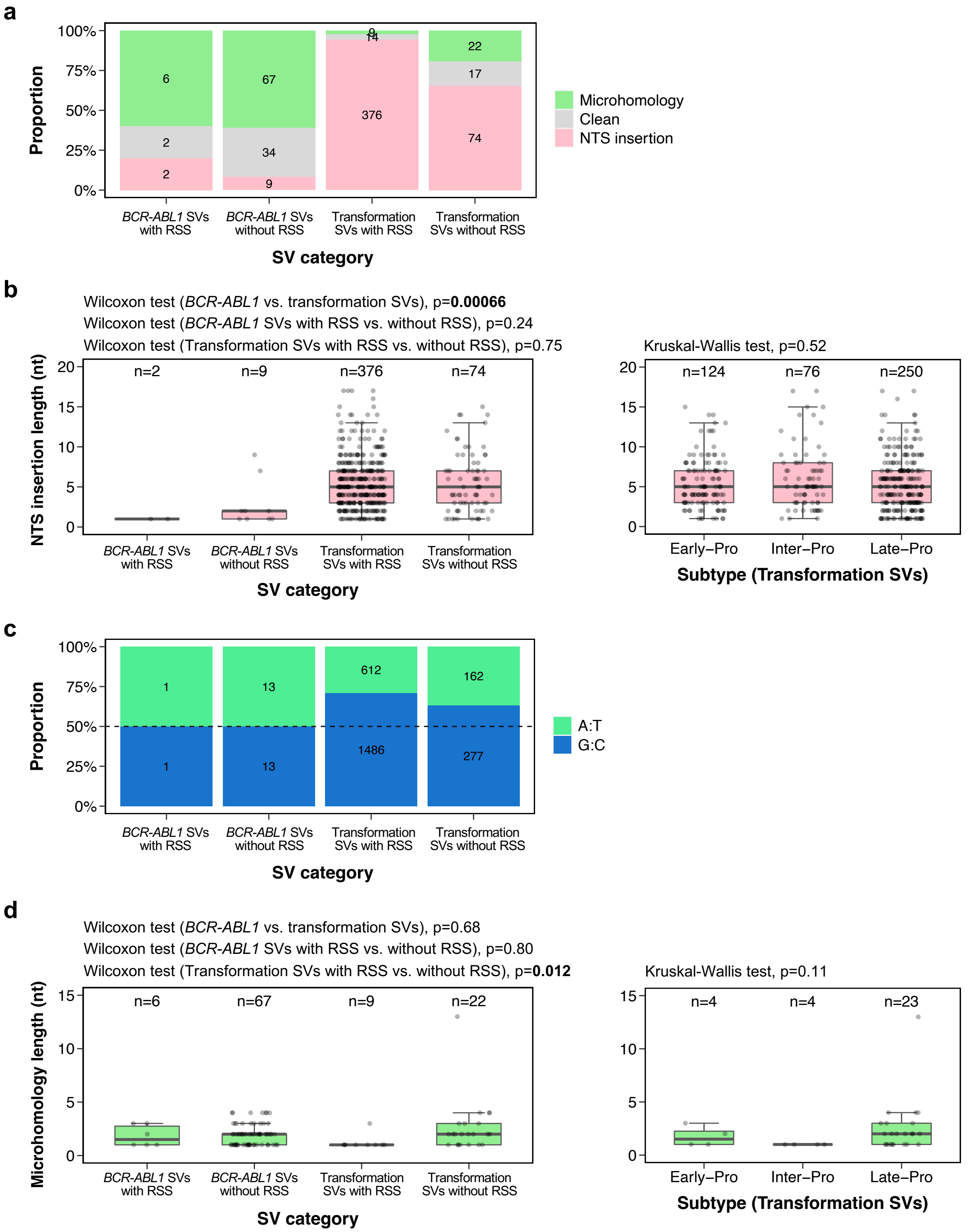

a, Proportions of SV junction types by SV category. **b**, NTS insertion lengths by SV category (left). NTS insertion lengths of transformation related SVs by molecular subtype (right). n represents number of SVs with NTS insertion. **c**, Proportions of G:C and A:T nucleotides in NTS insertions by SV category. **d**, Microhomology lengths by SV category (left). Microhomology lengths of transformation related SVs by molecular subtype (right). n represents number of SVs with microhomology.

Fig. S17 | Flow cytometry profiles of 4 diagnosis/relapse pairs

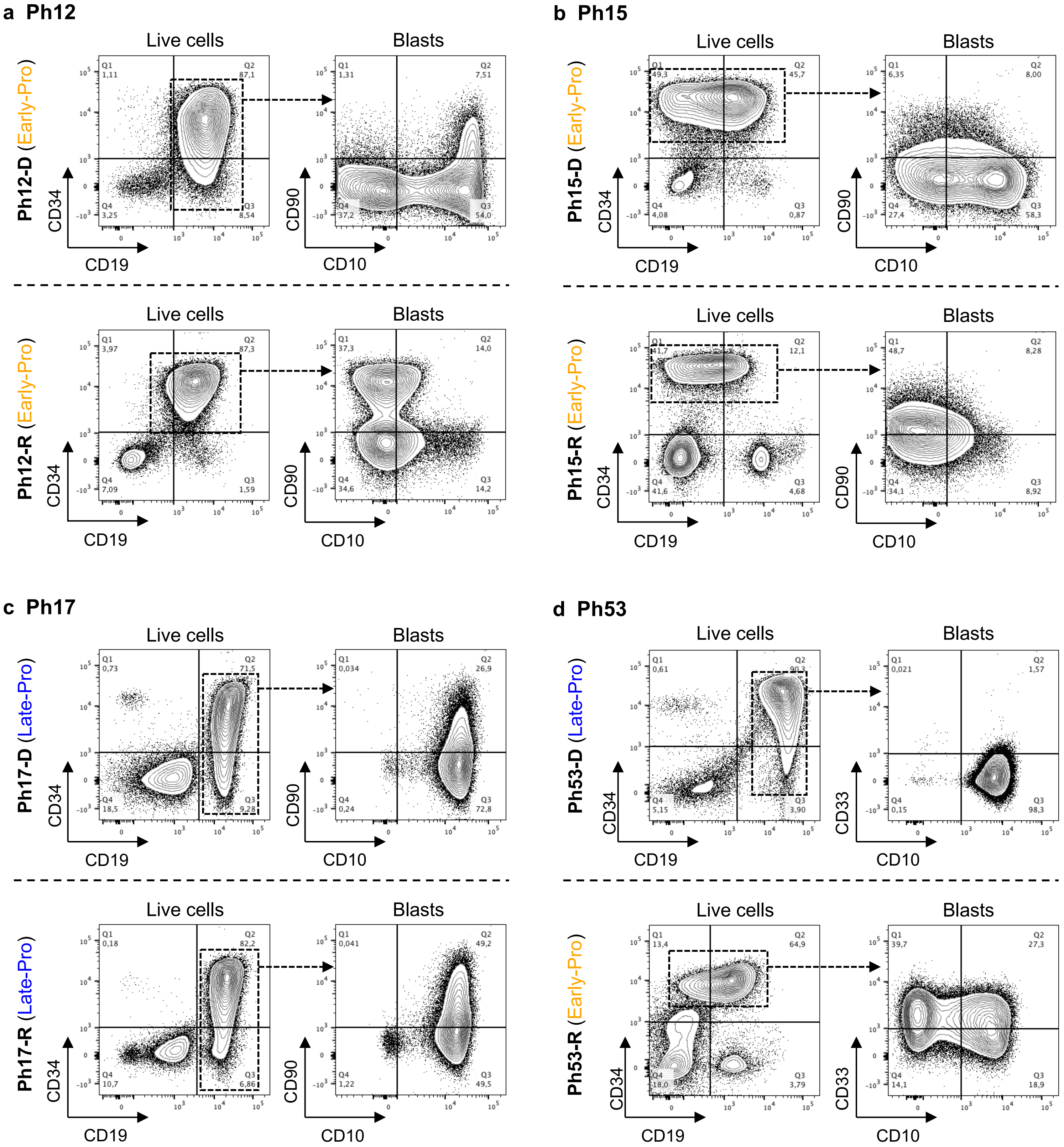

a-d, Immunophenotypic profiles of Ph12 (**a**), Ph15 (**b**), Ph17 (**c**), and Ph53 (**d**) at diagnosis (-D; top of each panel) and relapse (-R; bottom of each panel). Plots on the left display CD34/CD19 profiles of live cells with dashed boxes containing blasts. Plots on the right display CD90/CD10 or CD33/CD10 profiles of blasts.

Fig. S18 | Antigen receptor loci rearrangements in 4 diagnosis/relapse pairs

a Ph12

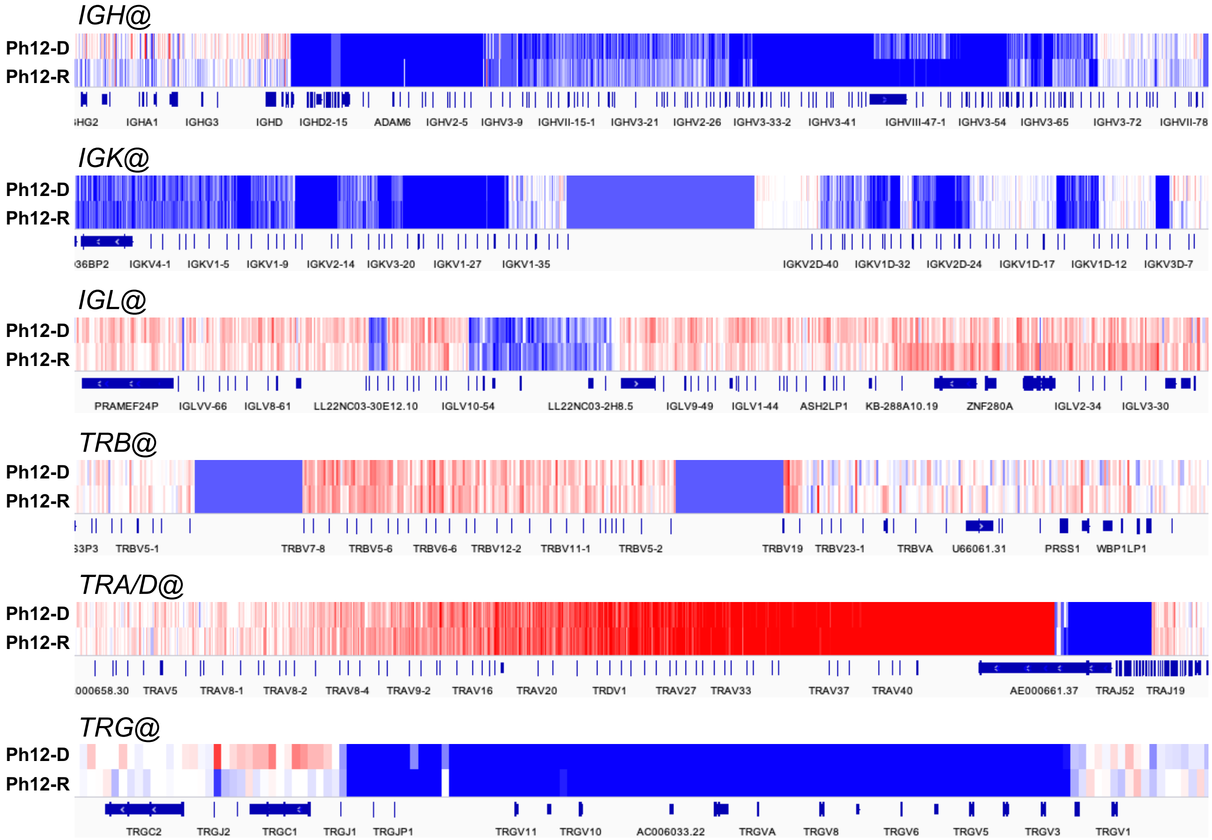

b Ph15

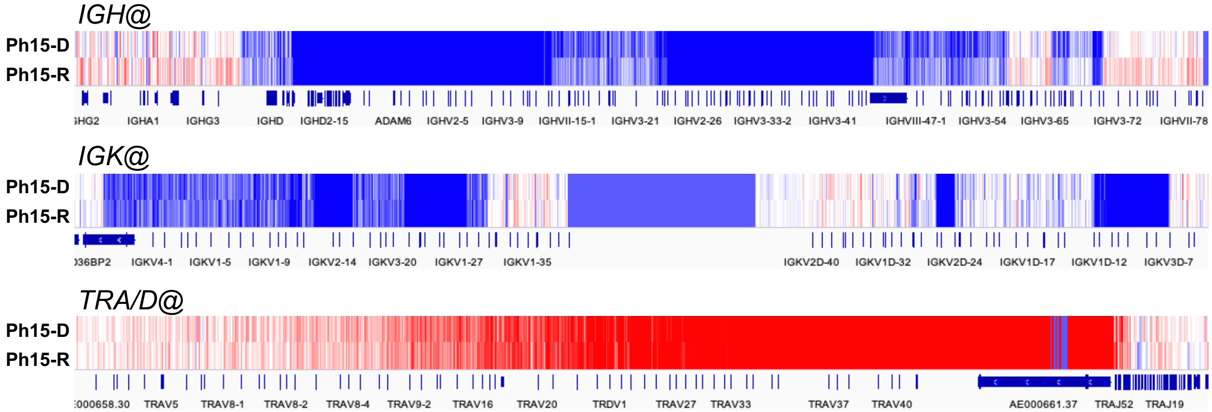

a-d, Copy number states across Ig and TCR loci visualized using the Integrative Genomics Viewer (IGV) for Ph12 (**a**), Ph15 (**b**), Ph17 (**c**), and Ph53 (**d**). Loci in germline (unrearranged) states are omitted. For each locus, diagnosis (-D) and relapse (-R) copy number states are shown. Blue represents copy number loss and red represents copy number gain relative to the reference T-cell genome. Ph12, Ph15, and Ph17 show no differences between diagnosis and relapse, whereas Ph53 shows differences at every locus.

Fig. S18 | Antigen receptor loci rearrangements in 4 diagnosis/relapse pairs (continued)

c Ph17

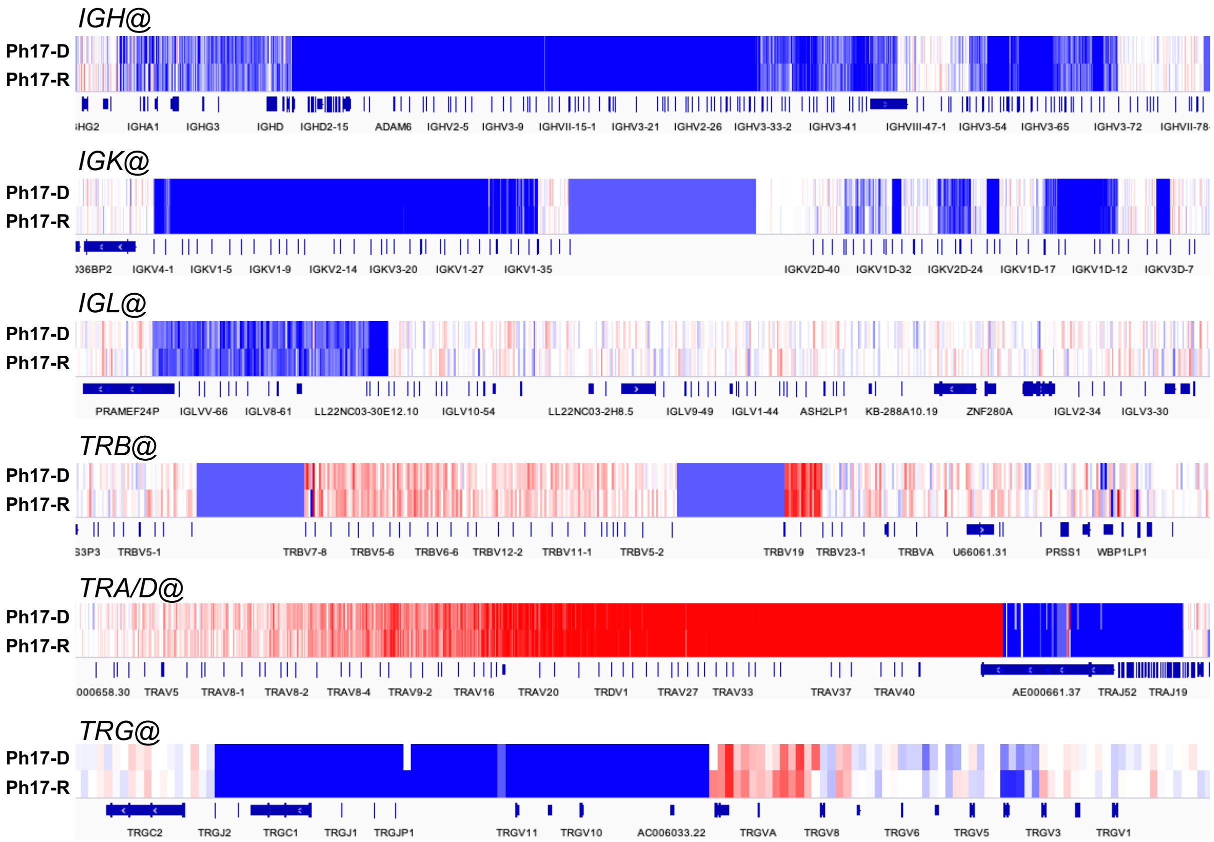

d Ph53

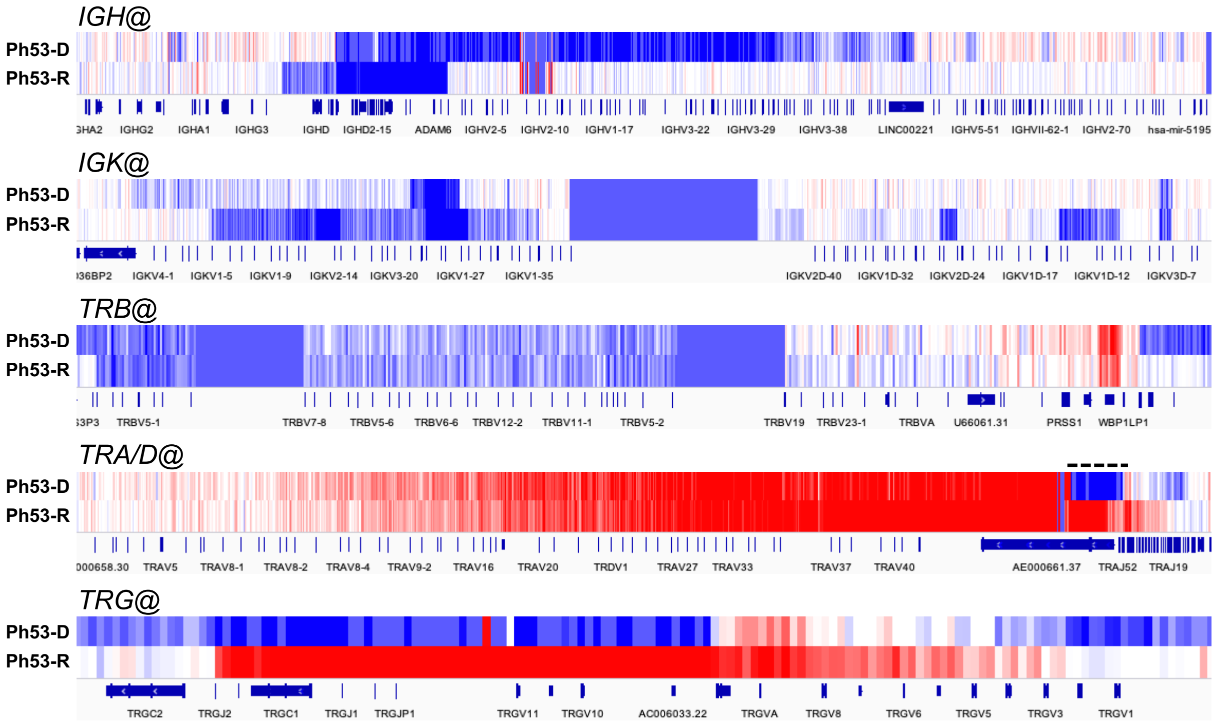

Fig. S19 | Model of disease progression in Ph53

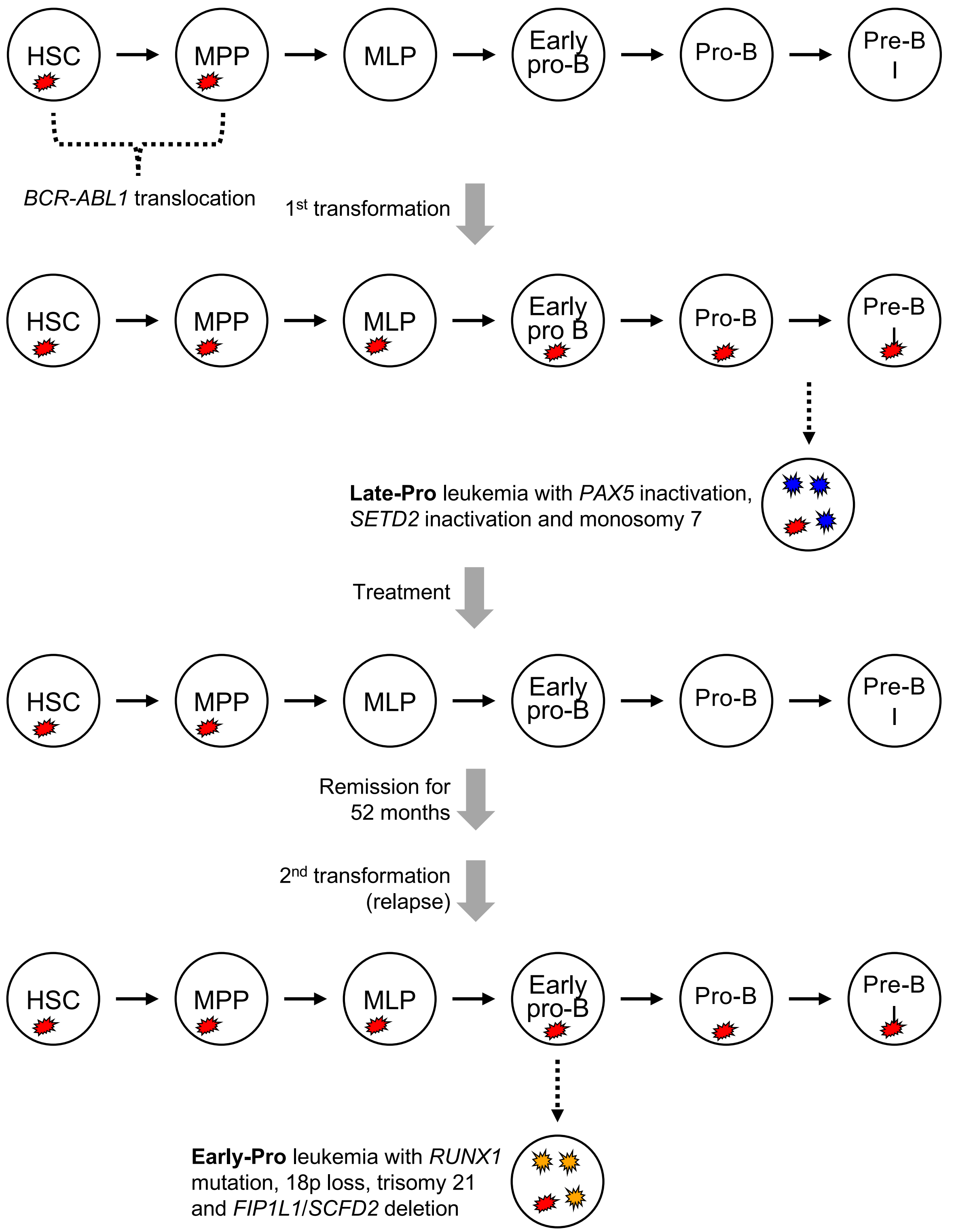

Model of a switch in phenotype from Late-Pro to Early-Pro leukemia for patient Ph53. *BCR-ABL1* translocation (red) arises in a multilineage stem cell (e.g. HSC or MPP) and is maintained from diagnosis to relapse. The diagnostic leukemia develops from acquisition of diagnosis-private alterations (blue) in potentially a pro-B clone. This leukemia is eradicated from the patient by therapy. After 52 months of remission, the relapse leukemia develops independently via acquisition of relapse-private alterations (orange) in an early pro-B clone.

Fig. S20 | Effect of gene copy number on survival

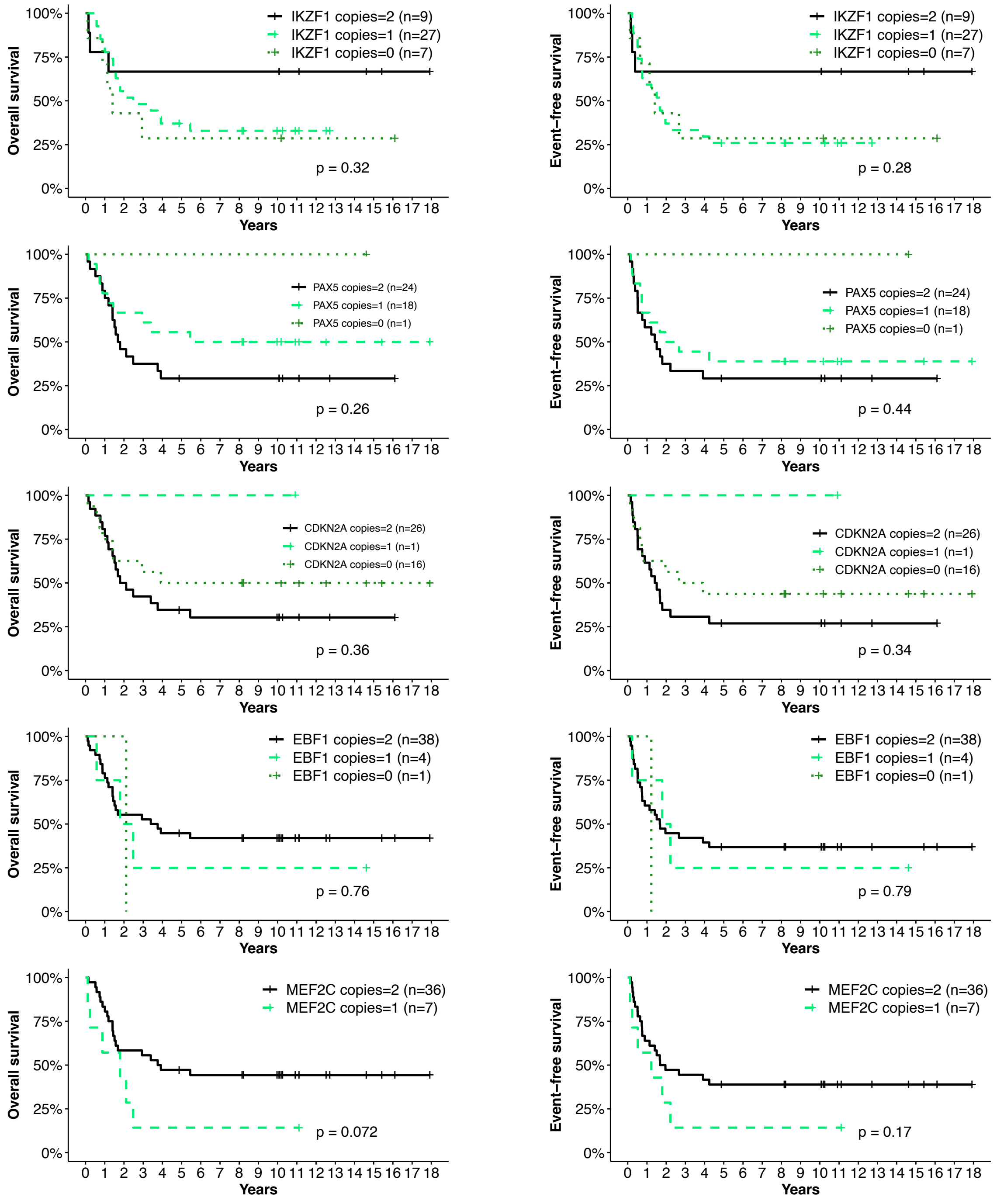

Effects of *IKZF1*, *PAX5*, *CDKN2A/B*, *EBF1*, and *MEF2C* gene copy number on patient survival. Inactivations are partitioned into heterozygous (gene copy=1) and homozygous (gene copy=0). Wildtypes have gene copy of 2. Kaplan-Meier estimates of overall survival (left) and event-free survival (right) are shown. Only the main cohort (n=43 for survival analysis) is analyzed since these patients have corresponding genomic data.

Fig. S21 | Effect of gene inactivation status on survival

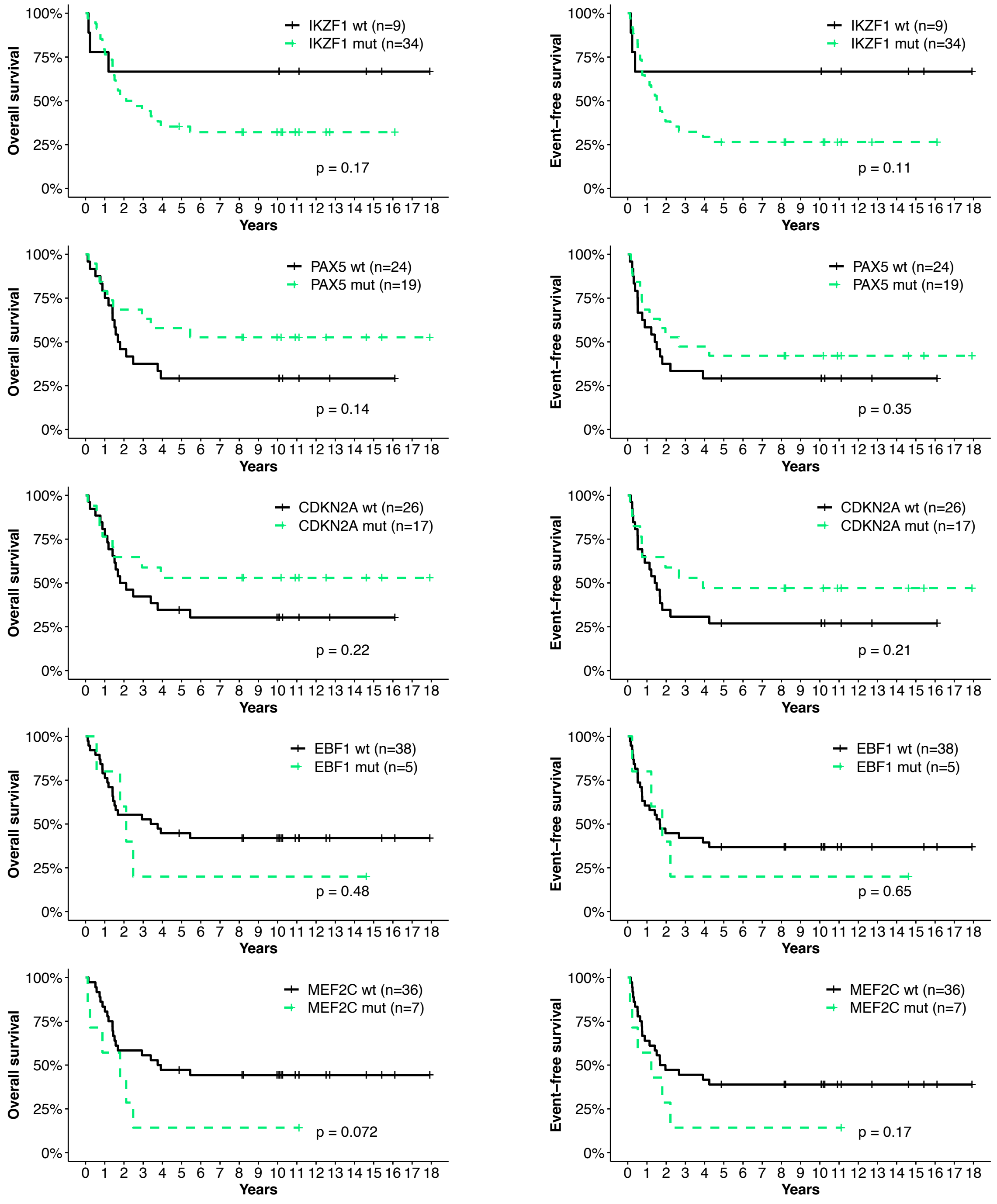

Effects of *IKZF1*, *PAX5*, *CDKN2A/B*, *EBF1*, and *MEF2C* gene inactivation (deletion or mutation) on patient survival. Kaplan-Meier estimates of overall survival (left) and event-free survival (right) are shown. Only the main cohort (n=43 for survival analysis) is analyzed since these patients have corresponding genomic data. wt, wildtype; mut, mutant/deleted.

Fig. S22 | Subtypes display survival differences even when patients with kinase domain mutations are excluded

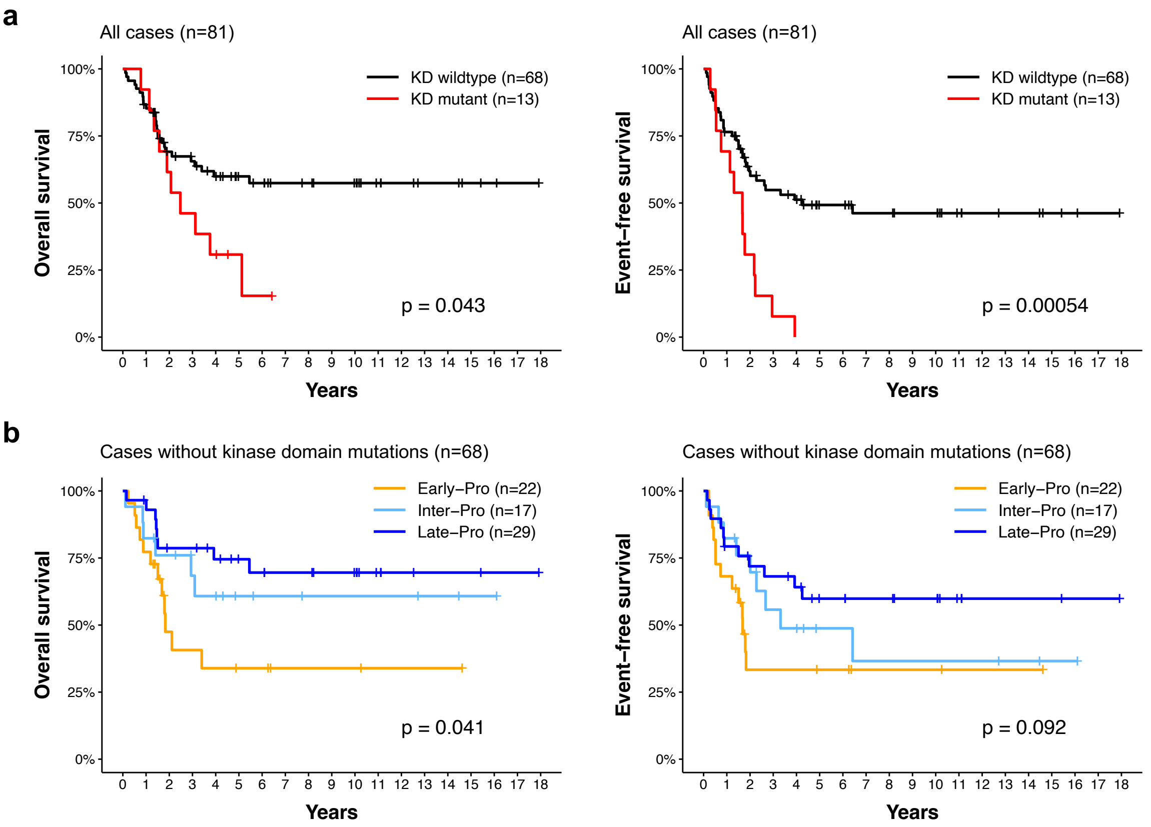

a, Kaplan-Meier estimates of overall survival (left) and event-free survival (right) by KD mutation status (n=81). **b**, Kaplan-Meier estimates of overall survival (left) and event-free survival (right) by molecular subtypes for patients who did not develop kinase domain mutations (n=68).

Fig. S23 | Cell cycle expression from single-cell RNA-seq

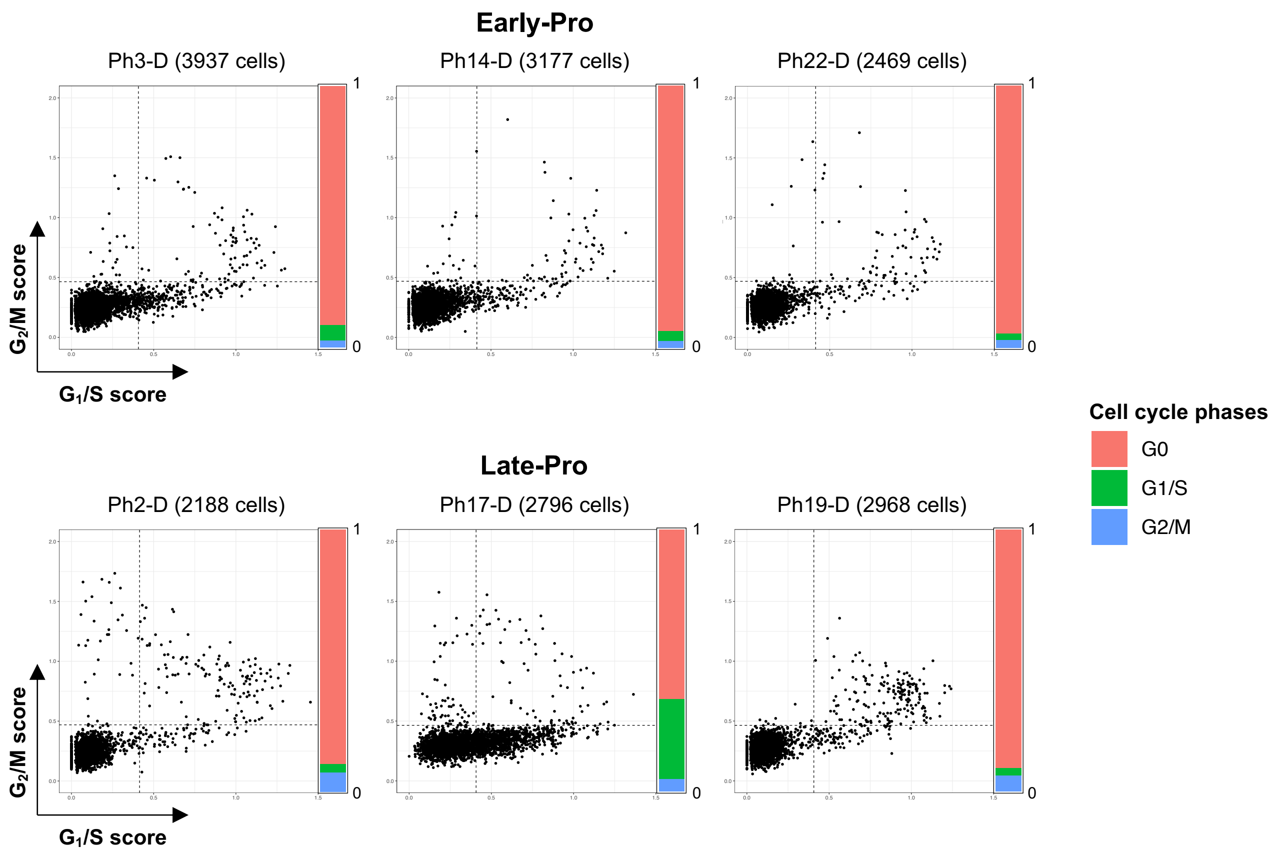

Average log2 expression (score) of G_1/S and G_2/M phase gene sets in single cells. Six cases (3 Early-Pro and 3 Late-Pro) not shown in Fig. 6c are shown here. Dashed lines denote the G_1/S and G_2/M cutoffs (one standard deviation greater than mean). Bars represent proportions of cells in G_0, G_1/S, and G_2/M phases for each sample.

Fig. S24 | Increased expression and phosphorylation of STAT5 in Early-Pro

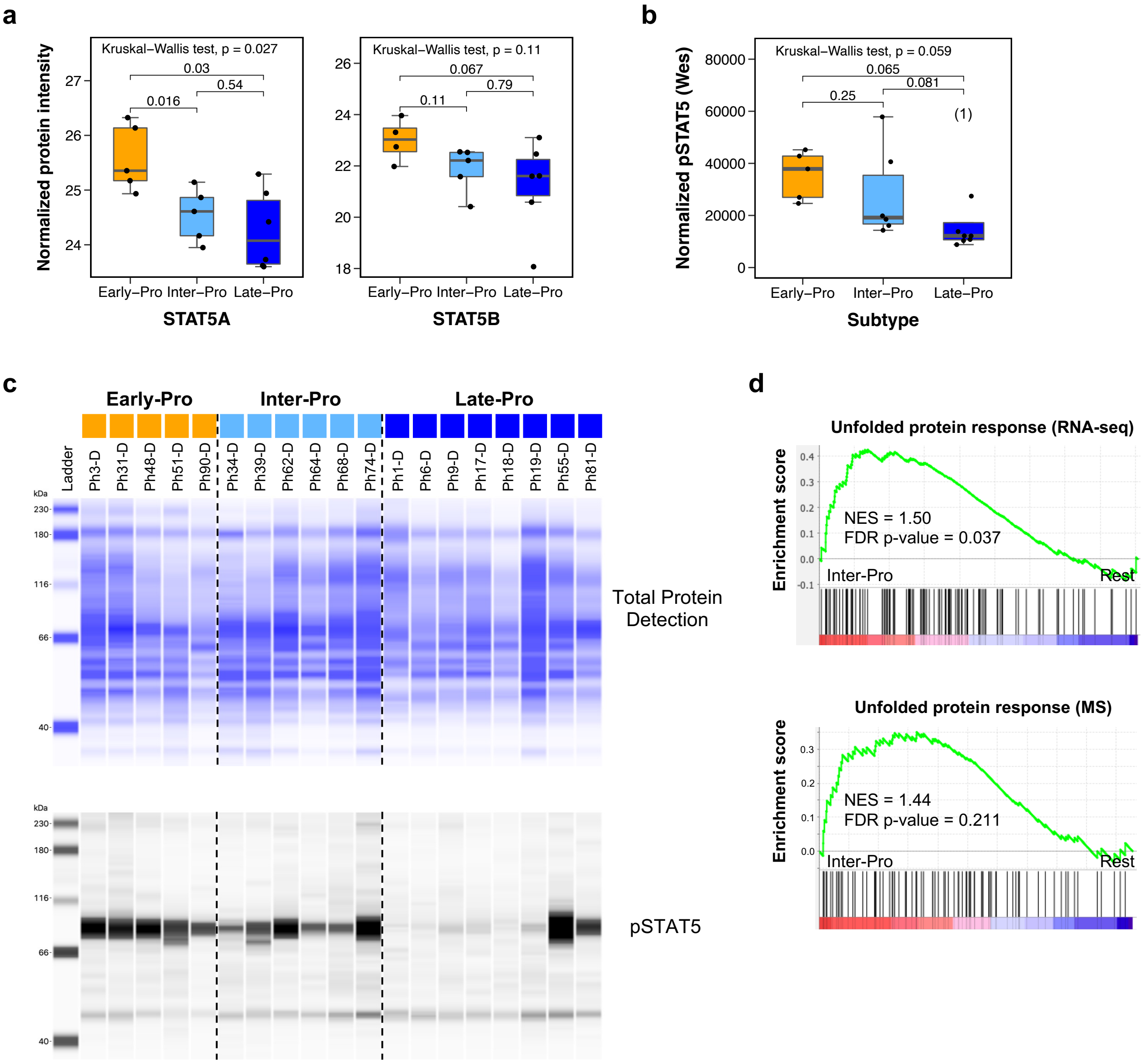

a, Normalized protein intensities of STAT5A and STAT5B from mass spectrometry (5 Early-Pro, 5 Inter-Pro, 6 Late-Pro leukemias). p-values from pairwise Wilcoxon rank-sum tests and Kruskal-Wallis test are shown. **b**, Phosphorylated STAT5 (pSTAT5 at Tyr694) levels normalized by total protein levels using the Wes immunodetection assay (5 Early-Pro, 6 Inter-Pro, 8 Late-Pro leukemias). p-values from pairwise Wilcoxon rank-sum tests and Kruskal-Wallis test are shown. Number in brackets indicate an outlier that is not shown to improve visibility of the overall comparison. **c**, Detection of total proteins (top) and pSTAT5 (bottom) by Wes. Top track displays the molecular subtype of each sample. **d**, Enrichment of unfolded protein response gene set in the Inter-Pro subtype (vs. rest) as detected by RNA-seq (top) and mass spectrometry (bottom).

Fig. S25 | Responses to 2nd/3rd generation TKI in 4 patients without kinase domain mutations

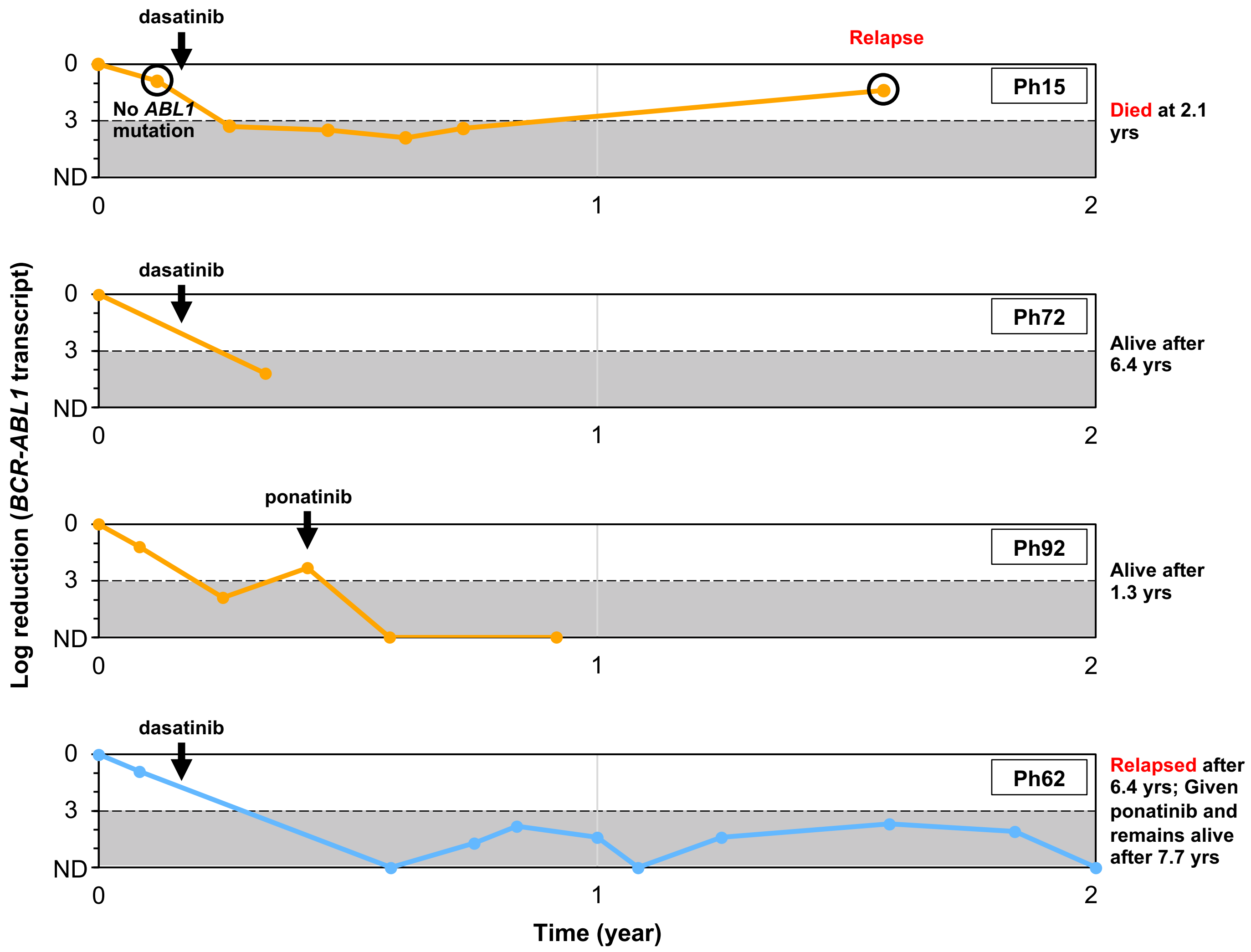

Residual disease plots for 4 patients (3 Early-Pro and 1 Inter-Pro) who received dasatinib or ponatinib after showing poor induction response. Each point represents a residual disease measurement, dashed line denotes log reduction of 3, and grey area denotes log reduction ≥3, which corresponds to major molecular response (MMR). Black circles in the Ph15 plot denote tests for kinase domain mutation, which were both negative. ND, not detected.

Fig. S26 | Comparison of CD34/CD19 expression profiles between subtypes

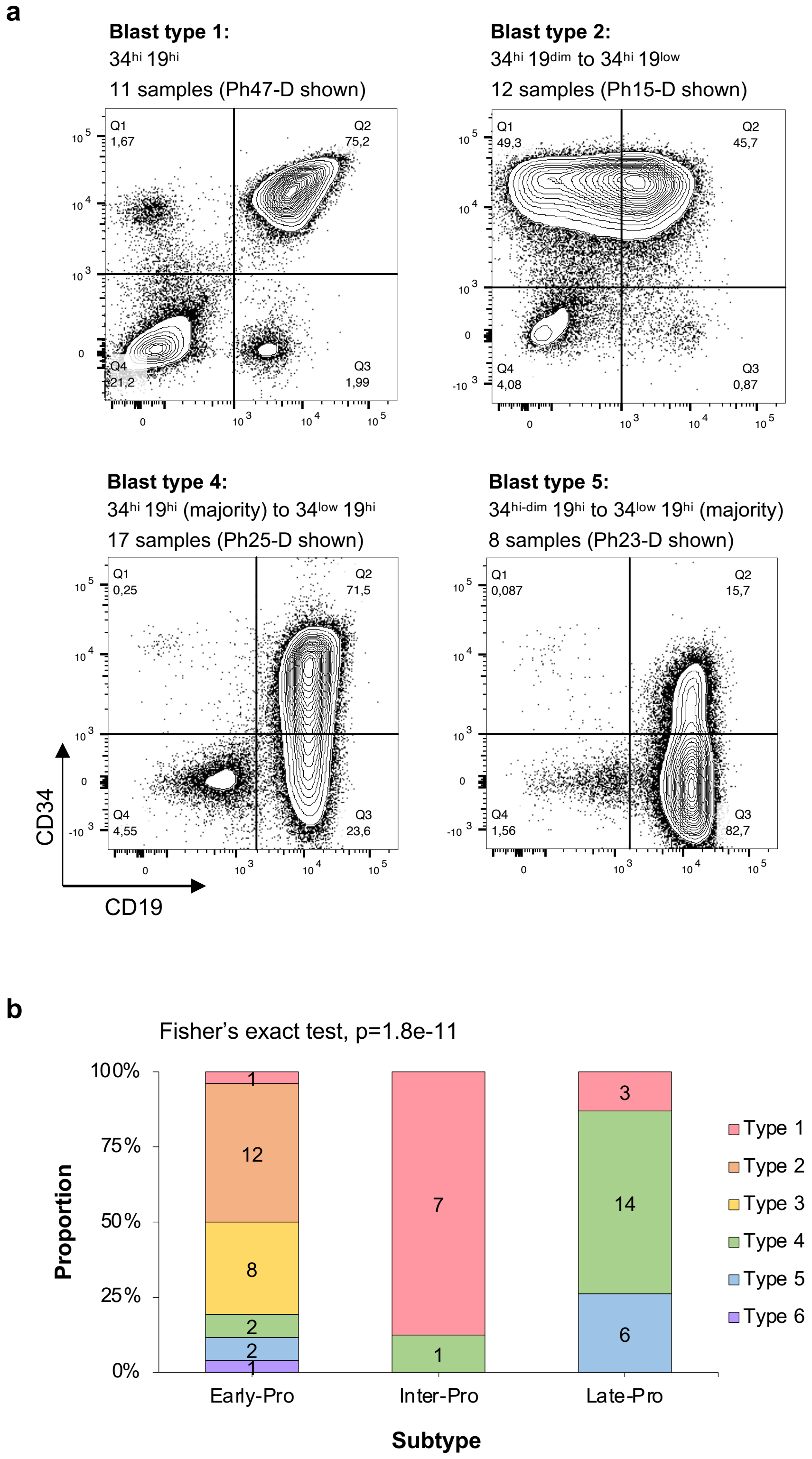

a, Six types of observed CD34/CD19 antigen expression profiles. An exemplar profile is shown for each blast type. **b**, Proportions of the six CD34/CD19 blast types by subtype. p-value from Fisher's exact test is shown.

Fig. S27 | Proportion of cells in each quadrant of CD34/CD19 expression

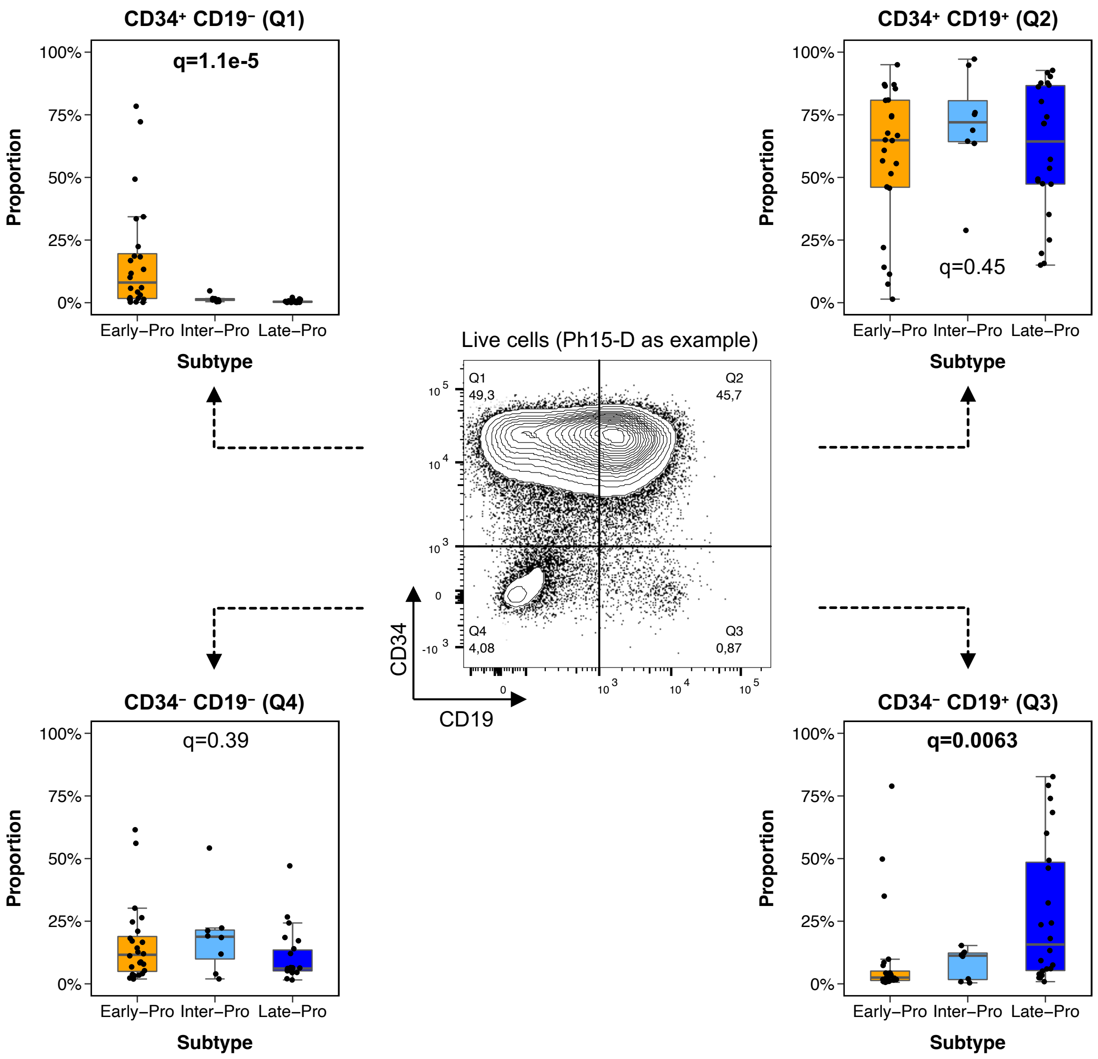

Proportions of live cells in four quadrants of CD34/CD19 profiles by subtype (23 Early-Pro, 8 Inter-Pro, 22 Late-Pro primary leukemias). FDR-adjusted p-values from Kruskal-Wallis test are shown. $CD34^+ CD19^-$ (Q1) and $CD34^- CD19^+$ (Q3) quadrants display statistically significant differences between subtypes.

Fig. S28 | Rearrangement patterns of the *IGH* locus

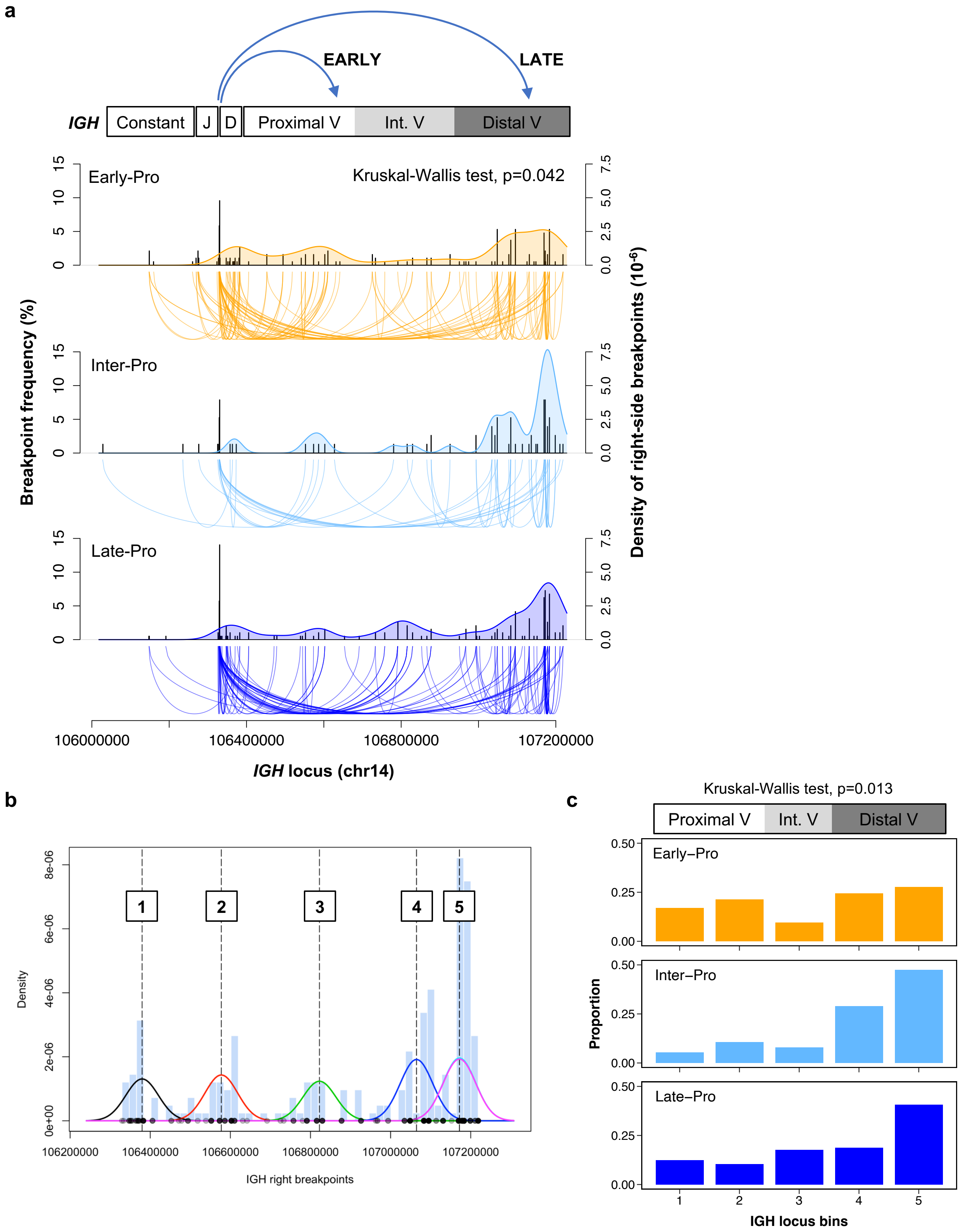

a, Top: Organization of the *IGH* locus in chr14. Constant region, J segments, D segments, and proximal/intermediate/distal V segments are shown in boxes. Early and late stage rearrangements are predicted to utilize proximal and distal V_H segments, respectively. Bottom: For each subtype, histogram of breakpoint positions (left axis) and density curve of rightside breakpoints (right axis) are shown. **b**, Five clusters/bins of all breakpoints from three subtypes. **c**, Proportions of rightside breakpoints in the five bins for each subtype.

Fig. S29 | Distribution of *BCR-ABL1* translocation breakpoints by disease type

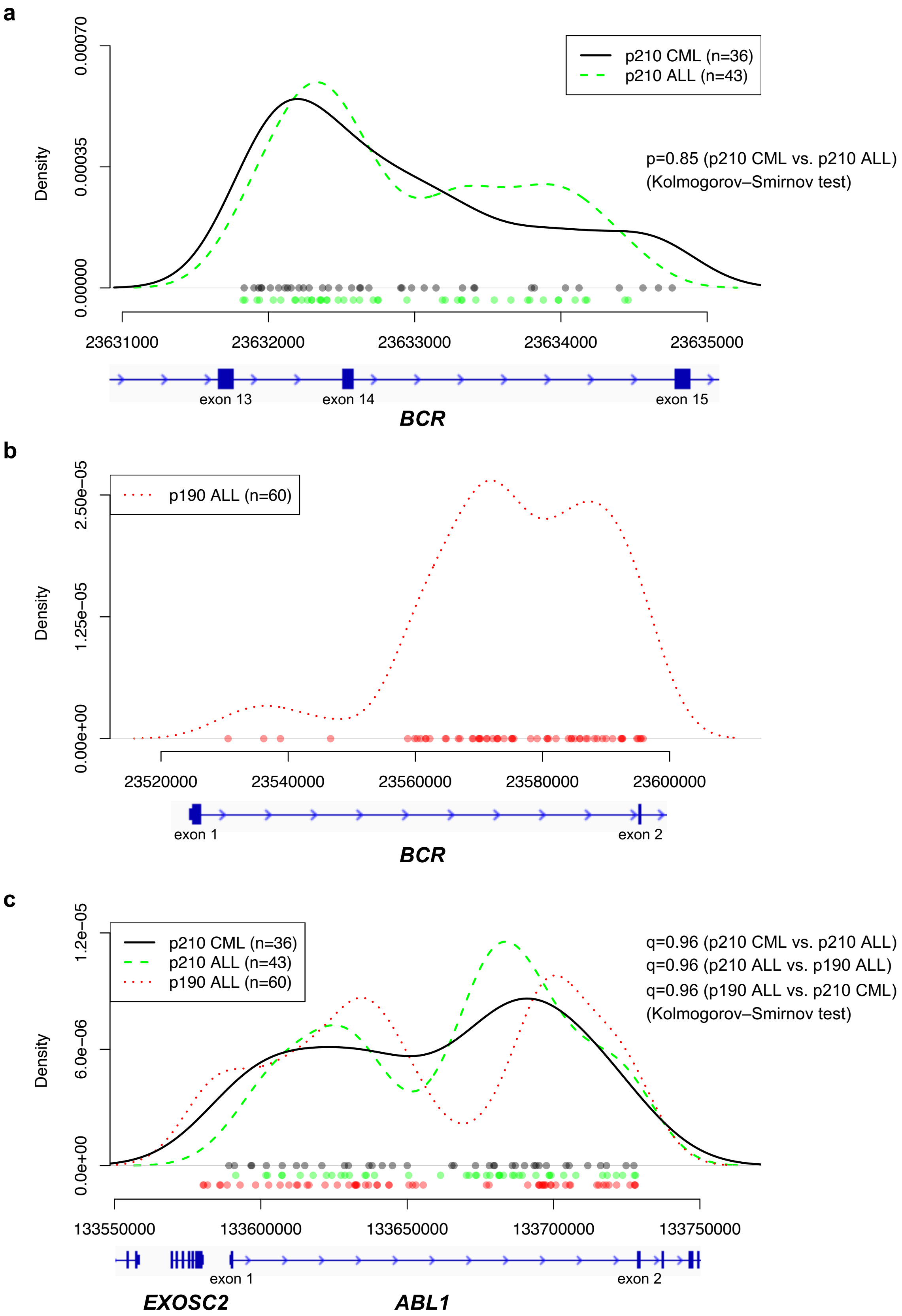

a-c, Distributions of *BCR-ABL1* translocation breakpoints between *BCR* exons 13 and 15 (p210 breakpoints) (**a**), between *BCR* exons 1 and 2 (p190 breakpoints) (**b**), and upstream of *ABL1* exon 2 (**c**). Breakpoint density curves are separated by disease types, p210 CML, p210 ALL and p190 ALL. *BCR-ABL1* rearrangements with nucleotide-level resolution from 3 datasets are used (n=139): this study (n=52), Score *et al.*[98] (n=78), and pediatric *BCR-ABL1* ALL samples from the EGAD00001000163 dataset (n=9). Points represent the breakpoint positions. p-value and FDR-adjusted p-values from Kolmogorov–Smirnov test are shown.

Fig. S30 | Distribution of *BCR-ABL1* translocation breakpoints by molecular subtype

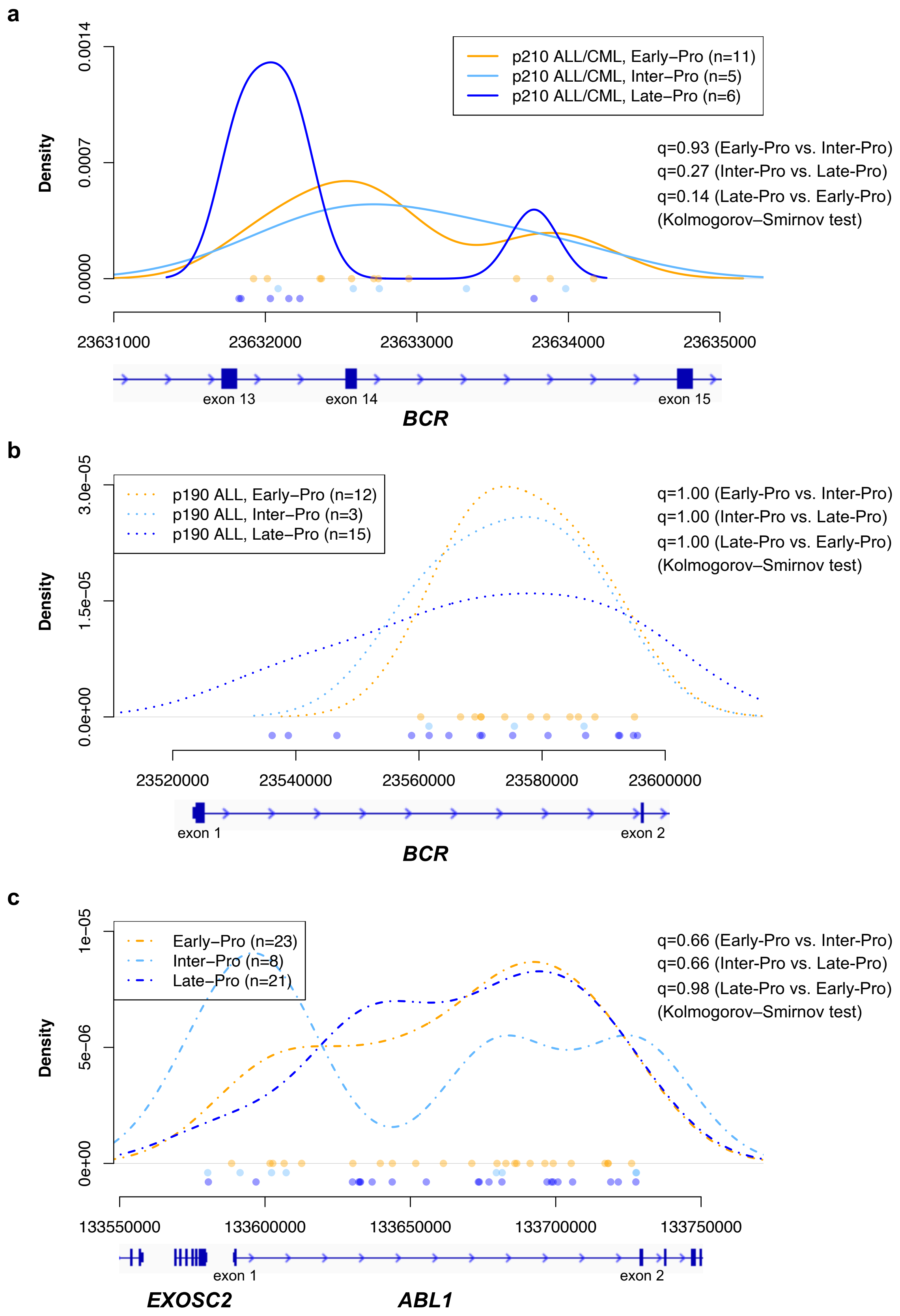

a-c, Distributions of *BCR-ABL1* translocation breakpoints between *BCR* exons 13 and 15 (p210 breakpoints) (**a**), between *BCR* exons 1 and 2 (p190 breakpoints) (**b**), and upstream of *ABL1* exon 2 (**c**). Breakpoint density curves are separated by molecular subtypes. *BCR-ABL1* rearrangements with nucleotide-level resolution from this study are used (n=52). Points represent the breakpoint positions. FDR-adjusted p-values from pairwise Kolmogorov–Smirnov test are shown.

Fig. S31 | Effect of *BCR-ABL1* isoforms or gain of Philadelphia chromosome on survival

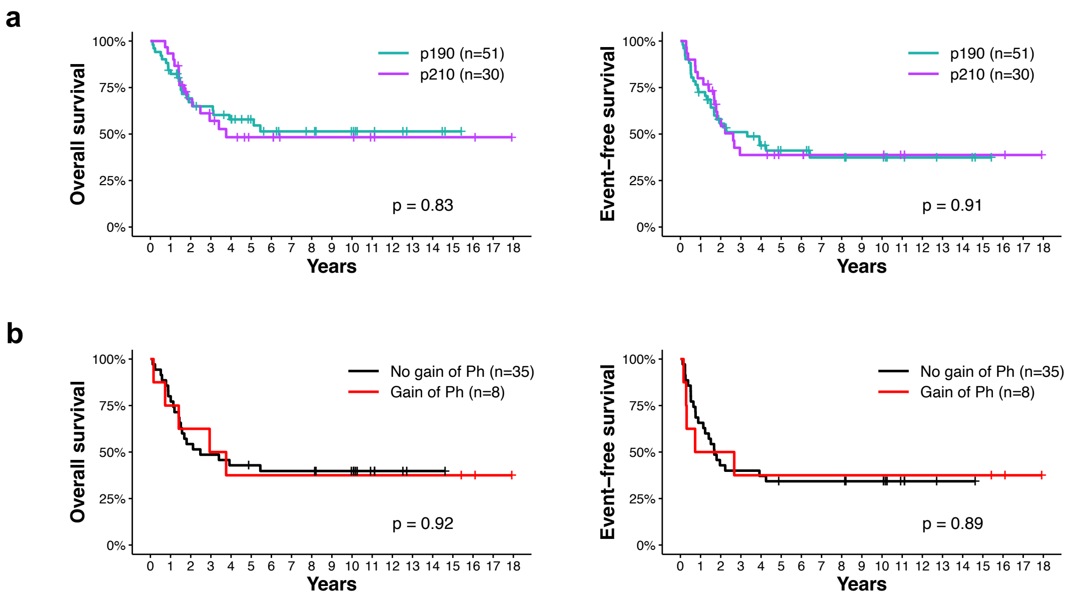

a, **b**, Effects of *BCR-ABL1* isoforms (p190/p210) (**a**) and gain of Philadelphia chromosome (**b**) on patient survival. Only the main cohort (n=43 for survival analysis) is used in **b** since patients in the other cohort do not have corresponding genomic data. Kaplan-Meier estimates of overall survival (left) and event-free survival (right) are shown. Ph, Philadelphia chromosome.

Fig. S32 | Comparison of SV types and sizes across SV categories

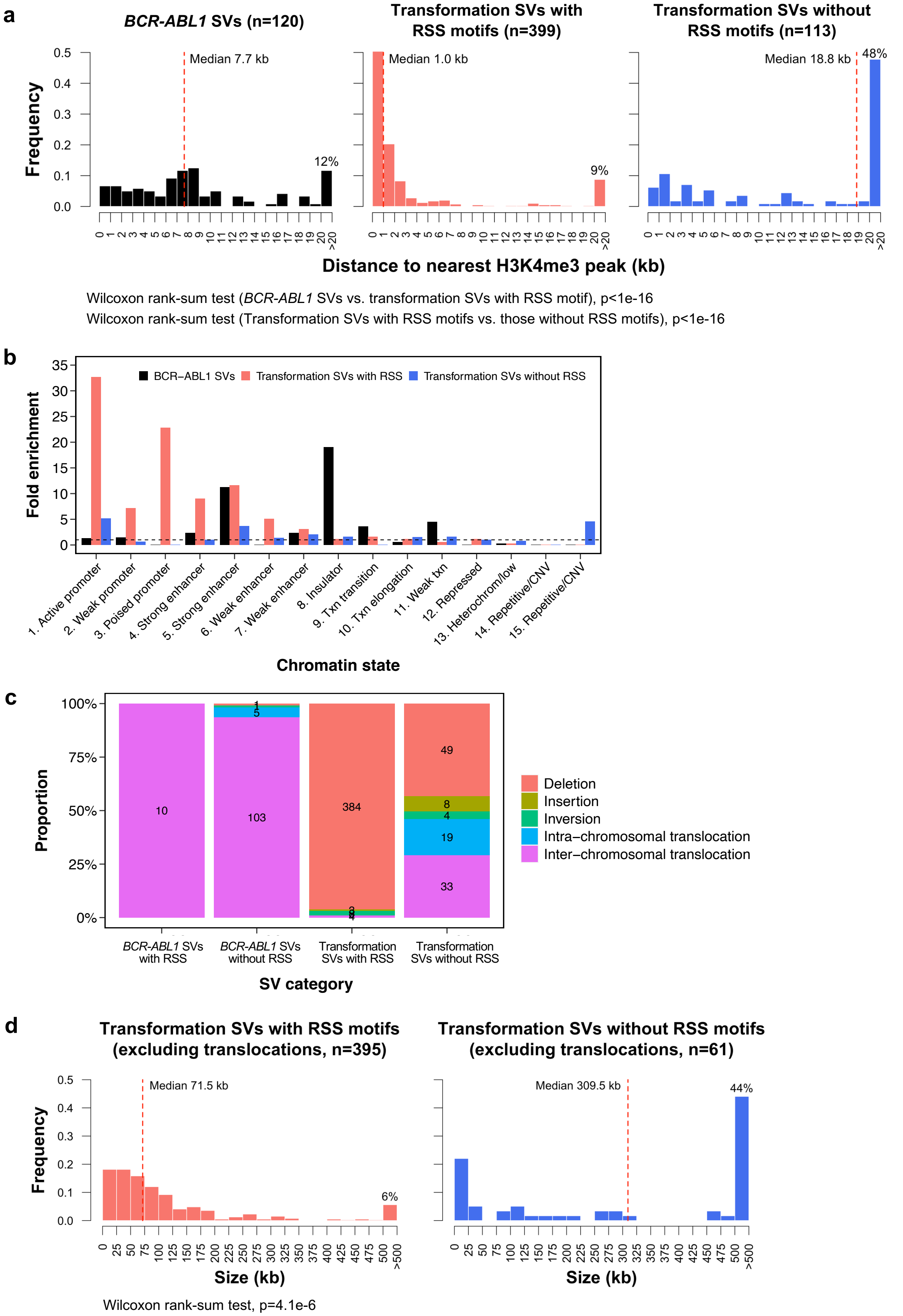

a, Histograms of distances between breakpoints and nearest H3K4me3 peaks for *BCR-ABL1* SVs (left), transformation SVs with RSS motifs (centre), and transformation SVs without RSS motifs (right). Red lines represent median sizes. **b**, Fold enrichment of 15 chromatin states in GM12878 for *BCR-ABL1* SVs, transformation SVs with RSS motifs, and transformation SVs without RSS motifs. Dashed line denotes fold enrichment of 1. **c**, Proportions of SV types in each SV category (*BCR-ABL1* or transformation related SVs with or without RSS motifs). Transformation SVs with RSS motifs are mostly deletions. **d**, Histograms of SV sizes for transformation SVs with RSS motifs (left) and those without RSS motifs (right). Only deletions, insertions, and inversions are considered for SV sizes. Red lines represent median sizes. Transformation SVs with RSS motifs are significantly smaller than those without RSS motifs.

Fig. S33 | Insertion of *IGK@* shard into *SLX4IP* deletion in Ph18-D

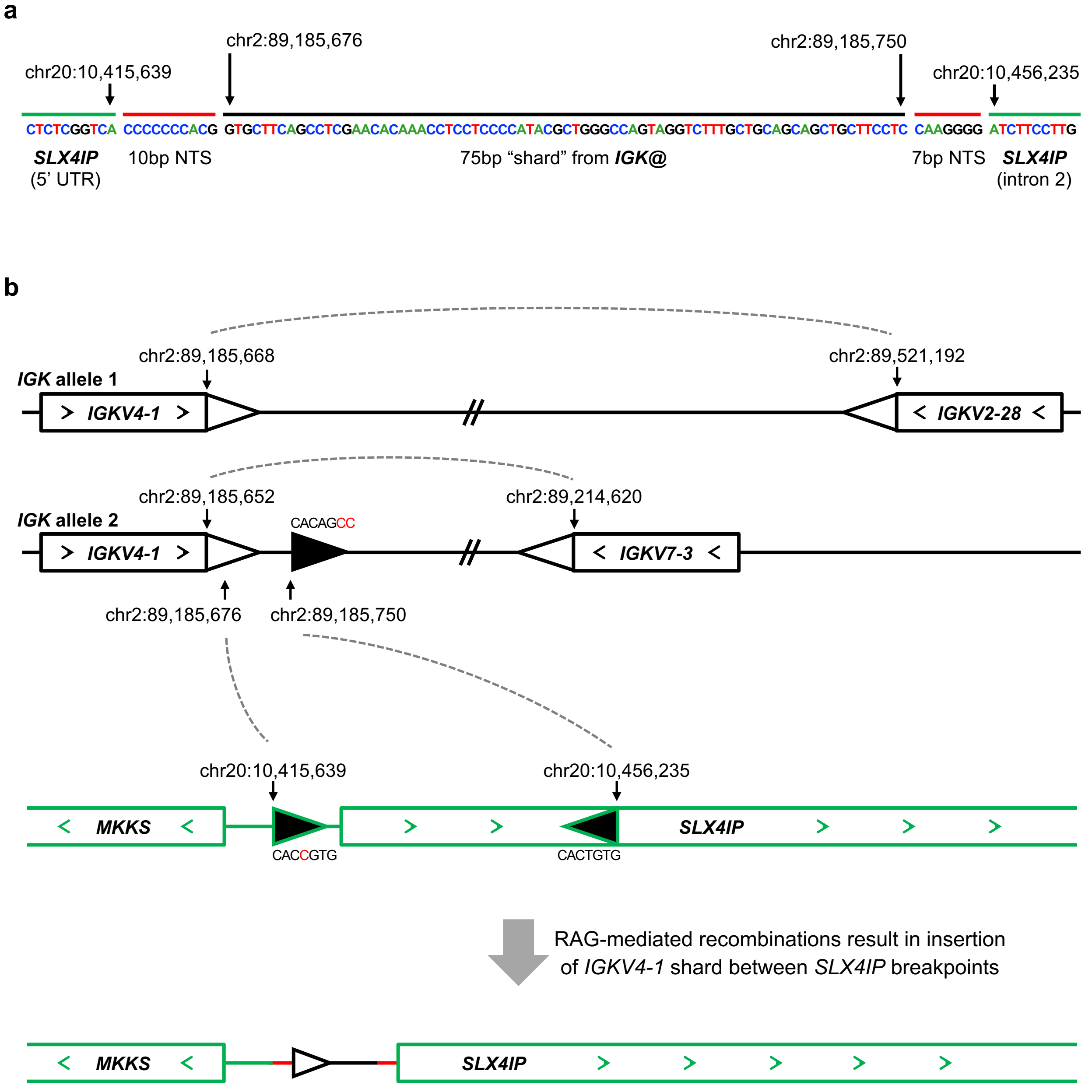

a, Nucleotide sequence between two breakpoints of *SLX4IP* deletion in Ph18-D. Green lines represent the *SLX4IP* gene, red lines represent NTS insertions, and a black line represents a 75 bp 'shard' from the *IGK* locus. **b**, Concurrence of *IGK@* rearrangements and *SLX4IP* deletion as a potential explanation for the insertion of *IGKV4-1* shard. When both alleles of *IGK@* are rearranged, a shard from the excised signal sequence of '*IGK* allele 2' was inserted into the *SLX4IP* deletion during RAG-mediated recombination. NTS was inserted between the shard and the *SLX4IP* gene at both ends by TdT. White triangles represent canonical RSS motifs, black triangles represent cryptic RSS motifs, and dashed lines represent RAG-mediated recombinations. Bases in cryptic RSS that deviate from the canonical RSS are highlighted in red font.

Fig. S34 | Number of RAG-mediated recombinations is strongly associated with *SLX4IP* deletion

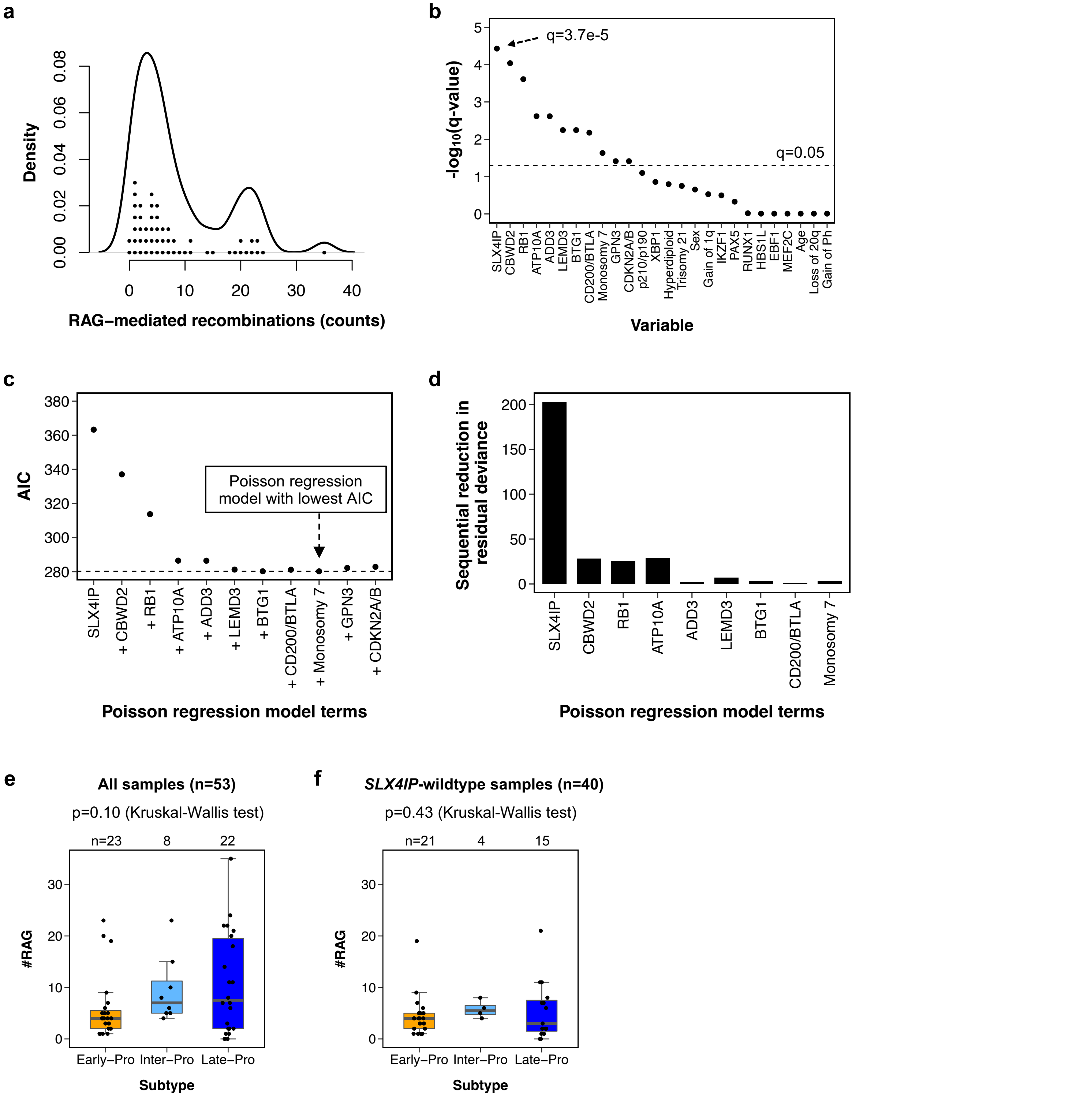

a, Bimodal distribution of numbers of RAG-mediated recombinations (#RAG) in *BCR-ABL1* lymphoblastic leukemia. **b**, Association between genetic and clinical markers and #RAG. Negative log10 of FDR-adjusted p-values from Wilcoxon rank-sum test are plotted. Dashed line denotes q=0.05. **c**, Poisson regression model to identify the best predictor of #RAG. AIC (Akaike information criterion) values from models fitted via sequential addition of 11 genetic markers are shown. A model using the top nine markers resulted in the model with lowest AIC. **d**, Sequential reduction in residual deviance in the nine-marker predictor. **e**, #RAG by subtype for all primary leukemias. **f**, #RAG by subtype for *SLX4IP*-wildtype primary leukemias.

Fig. S35 | Detection of low-frequency mutations in *ABL1* kinase domain using SimSen-seq

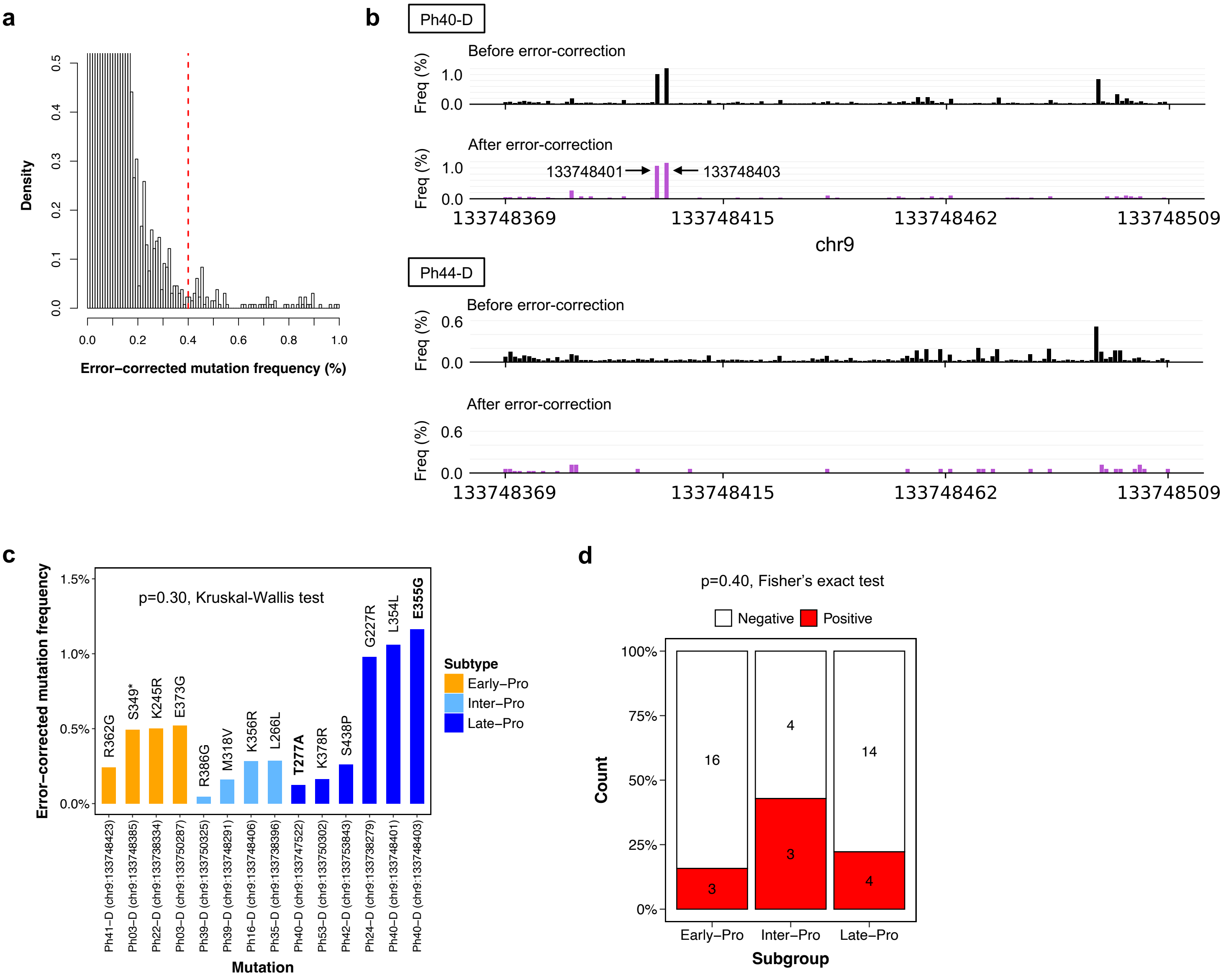

a, Histogram of SimSen-seq non-reference mutation frequency after error-correction using a minimum molecular barcode family size of 20. A cutoff is set at 0.4% (Methods). **b**, Exemplar low-frequency mutations in the *ABL1* kinase domain in Ph40-D (top). Two mutations at chr9:133748401 and chr9:133748403 remain after error-correction and are not detected in other samples, such as Ph44-D (bottom). **c**, Error-corrected mutation frequency of 14 variants detected by SimSen-seq. Kruskal-Wallis test was used to compare mutation frequencies between subtypes. **d**, Proportions of leukemias in each subtype with and without low-frequency mutations in the *ABL1* kinase domain. p-value is from Fisher's exact test.

Supplementary references

92. van Dongen, J. J. & Wolvers-Tettero, I. L. Analysis of immunoglobulin and T cell receptor genes. Part II: Possibilities and limitations in the diagnosis and management of lymphoproliferative diseases and related disorders. *Clin. Chim. Acta* **198**, 93–174 (1991).

93. Mejstrikova, E. *et al.* Prognosis of children with mixed phenotype acute leukemia treated on the basis of consistent immunophenotypic criteria. *Haematologica* **95**, 928–935 (2010).

94. Nacheva, E. P. *et al.* Deletions of immunoglobulin heavy chain and T cell receptor gene regions are uniquely associated with lymphoid blast transformation of chronic myeloid leukemia. *BMC Genomics* **11**, 41 (2010).

95. Gerasimova, T. *et al.* A structural hierarchy mediated by multiple nuclear factors establishes *IgH* locus conformation. *Genes Dev.* **29**, 1683–1695 (2015).

96. Jhunjhunwala, S. *et al.* The 3D Structure of the Immunoglobulin Heavy-Chain Locus: Implications for Long-Range Genomic Interactions. *Cell* **133**, 265–279 (2008).

97. Scrucca, L., Fop, M., Murphy, T. B. & Raftery, A. E. mclust 5: Clustering, Classification and Density Estimation Using Gaussian Finite Mixture Models. *R J* **8**, 289–317 (2016).

98. Score, J. *et al.* Analysis of genomic breakpoints in p190 and p210 BCR-ABL indicate distinct mechanisms of formation. *Leukemia* **24**, 1742–1750 (2010).

99. Matthews, A. G. W. *et al.* RAG2 PHD finger couples histone H3 lysine 4 trimethylation with V(D)J recombination. *Nature* **450**, 1106–1110 (2007).

100. Kirkham, C. M. *et al.* Cut-and-Run: A distinct mechanism by which V(D)J recombination causes genome instability. *Mol. Cell* **74**, 584-597.e9 (2019).

101. Roberts, K. G. *et al.* Targetable kinase-activating lesions in Ph-like acute lymphoblastic leukemia. *N. Engl. J. Med.* **371**, 1005–1015 (2014).

102. Svendsen, J. M. *et al.* Mammalian BTBD12/SLX4 assembles a Holliday junction resolvase and is required for DNA repair. *Cell* **138**, 63–77 (2009).

103. Panier, S. *et al.* SLX4IP antagonizes promiscuous BLM activity during ALT maintenance. *Mol. Cell* **76**, 27-43.e11 (2019).

104. Schatz, D. G. & Ji, Y. Recombination centres and the orchestration of V(D)J recombination. *Nat. Rev. Immunol.* **11**, 251–263 (2011).

105. Short, N. J. *et al.* Ultra-accurate Duplex Sequencing for the assessment of pretreatment ABL1 kinase domain mutations in Ph+ ALL. *Blood Cancer J.* **10**, 61 (2020).

106. Soverini, S. *et al.* Unraveling the complexity of tyrosine kinase inhibitor-resistant populations by ultra-deep sequencing of the BCR-ABL kinase domain. *Blood* **122**, 1634–1648 (2013).

107. Apperley, J. F. Part I: mechanisms of resistance to imatinib in chronic myeloid leukaemia. *Lancet Oncol.* **8**, 1018–1029 (2007).
