# Supplementary material for: Transcriptomic classes of BCR-ABL1 lymphoblastic leukemia
Source: Nat Genet. 2023 Jun 19;55(7):1186–97. doi: 10.1038/s41588-023-01429-4 (PMC10335939; doi:10.1038/s41588-023-01429-4)
Supplement: Supplementary file 2 — Reporting Summary [file 41588_2023_1429_MOESM2_ESM.pdf]

nature portfolio

Corresponding author(s): Faiyaz Notta

Last updated by author(s): Apr 21, 2023

Reporting Summary

Nature Portfolio wishes to improve the reproducibility of the work that we publish. This form provides structure for consistency and transparency in reporting. For further information on Nature Portfolio policies, see our Editorial Policies and the Editorial Policy Checklist.

Statistics

For all statistical analyses, confirm that the following items are present in the figure legend, table legend, main text, or Methods section.

n/a	Confirmed	
☐	☒	The exact sample size (*n*) for each experimental group/condition, given as a discrete number and unit of measurement
☐	☒	A statement on whether measurements were taken from distinct samples or whether the same sample was measured repeatedly
☐	☒	The statistical test(s) used AND whether they are one- or two-sided<br>*Only common tests should be described solely by name; describe more complex techniques in the Methods section.*
☐	☒	A description of all covariates tested
☐	☒	A description of any assumptions or corrections, such as tests of normality and adjustment for multiple comparisons
☐	☒	A full description of the statistical parameters including central tendency (e.g. means) or other basic estimates (e.g. regression coefficient) AND variation (e.g. standard deviation) or associated estimates of uncertainty (e.g. confidence intervals)
☐	☒	For null hypothesis testing, the test statistic (e.g. *F*, *t*, *r*) with confidence intervals, effect sizes, degrees of freedom and *P* value noted<br>*Give P values as exact values whenever suitable.*
☒	☐	For Bayesian analysis, information on the choice of priors and Markov chain Monte Carlo settings
☐	☒	For hierarchical and complex designs, identification of the appropriate level for tests and full reporting of outcomes
☐	☒	Estimates of effect sizes (e.g. Cohen's *d*, Pearson's *r*), indicating how they were calculated

Our web collection on statistics for biologists contains articles on many of the points above.

Software and code

Policy information about availability of computer code

Data collection	Not applicable
Data analysis	R v3.6.1, STAR v2.5.2a, HTSeq v0.9.1, DEseq2 v1.18.1, sva v3.42.0, ConsensusClusterPlus v1.40.0, GSEA Prerank v4.1.0, STAR-Fusion v1.0.0, MiXCR v3.0.3, CellRanger v3.0.1, Seurat v3.1.2, SingleR v1.0.6, Monocle 3 v0.2.3.0, Symphony v0.1.1, bwa v0.7.12, Picard v1.90, Genome Analysis Tool Kit (GATK) v3.6.0, Strelka v1.0.7, MuTect v1.1.4, HMMcopy v0.1.1, CREST alpha, Delly v0.5.5, Debarcer, COMPASS v4.0.0, MaxQuant v1.6.3.3, and WebLogo v2.8.2

For manuscripts utilizing custom algorithms or software that are central to the research but not yet described in published literature, software must be made available to editors and reviewers. We strongly encourage code deposition in a community repository (e.g. GitHub). See the Nature Portfolio guidelines for submitting code & software for further information.

Data

Policy information about availability of data

All manuscripts must include a data availability statement. This statement should provide the following information, where applicable:

- Accession codes, unique identifiers, or web links for publicly available datasets
- A description of any restrictions on data availability
- For clinical datasets or third party data, please ensure that the statement adheres to our policy

Whole genome sequencing and RNA-seq data are deposited and publicly available on the European Genome-Phenome Archive (EGAS00001007167). Mass spectrometry data can be found in Supplementary Table 14.

Human research participants

Policy information about studies involving human research participants and Sex and Gender in Research.

Reporting on sex and gender	Biological sex is used throughout the paper and sex proportions of cohorts and leukemia subtypes are provided. In 93 patients, 53 were male and 40 were female.
Population characteristics	In the combined cohort, 93 diagnostic (treatment-naive) samples and 4 relapse samples were studied. Median age of the cohort is 51 y.o. (range 13~88 y.o.). 86 patients were diagnosed with B-cell acute lymphoblastic leukemia, 5 with chronic myeloid leukemia in lymphoid blast crisis, and 2 with mixed-phenotype acute leukemia.
Recruitment	We studied BCR-ABL1 lymphoblastic leukemia samples from the UHN Leukemia Biobank in Toronto, Canada. The first 27 samples were selected to have equal proportions of p190 and p210 isoforms of BCR-ABL1 fusion, and the remaining samples were randomly selected. Because the initial observation, and part of the final conclusion, of this study was that transcriptomic classes of BCR-ABL1 ALL are not driven by BCR-ABL1 isoforms, we believe this initial selection process has no impact on the results. This is also mentioned in Methods.
Ethics oversight	The study was approved by the Research Ethics Board of the University Health Network (REB# 01-0573) and written informed consent was obtained from all patients.

Note that full information on the approval of the study protocol must also be provided in the manuscript.

Field-specific reporting

Please select the one below that is the best fit for your research. If you are not sure, read the appropriate sections before making your selection.

☒ Life sciences ☐ Behavioural & social sciences ☐ Ecological, evolutionary & environmental sciences

For a reference copy of the document with all sections, see nature.com/documents/nr-reporting-summary-flat.pdf

Life sciences study design

All studies must disclose on these points even when the disclosure is negative.

Sample size	Samples from 93 patients with BCR-ABL1 lymphoblastic leukemia were obtained from the University Health Network Leukemia Biobank, who collected them between 1992 and 2019. This disease is rare, and based on our initial observation of two broad phenotypes - mixed-lineage and B-cell precursor - we deemed the sample size sufficient. Sample sizes for single-cell RNA-seq (n=9), colony formation assay (n=14), and mass spectrometry (n=16) were determined by availability of resources and frozen cells/pellets. For these experiments, every effort was made to maintain the observed proportions of leukemia subtypes.
Data exclusions	All patient samples were included in analyses except for the following. Survival analyses were performed after excluding 12 out of 93 patients; 5 were CML cases in lymphoid blast crisis, 5 were not given a TKI at induction, and 2 died within 30 days of diagnosis. For analysis of clinical flow cytometry data, most samples had antigen expression data available, but a small subset did not have data available for certain antigens.
Replication	To confirm our findings, we investigated two patient cohorts through different experimental approaches. Samples from the main cohort (n=53) were purified by FACS and subjected to whole-transcriptome sequencing, whereas samples from the second cohort (n=40) were purified by magnetic separation and subjected to 3' RNA-seq. Transcriptomic clusters identified from the main cohort were replicated in the second cohort. Subsequent experiments, such as single-cell RNA-seq and colony formation assay, were performed with multiple samples from each leukemia subtype when possible (i.e. biological replicates) but were not repeated.
Randomization	In this retrospective study, we randomly selected BCR-ABL1 lymphoblastic leukemia samples stored at the University Health Network Leukemia Biobank without any prior knowledge regarding sex, age, treatment responses, outcomes, flow cytometry findings, or molecular findings (except for detection BCR-ABL1) of the patients. Data from this study were not used to alter the clinical treatment of the patients.

Blinding | This is not relevant to our study as there was no group allocation. After molecular subtypes were identified, sample IDs were known to the investigators to keep track of samples used in each experiment/analysis.

Reporting for specific materials, systems and methods

We require information from authors about some types of materials, experimental systems and methods used in many studies. Here, indicate whether each material, system or method listed is relevant to your study. If you are not sure if a list item applies to your research, read the appropriate section before selecting a response.

Materials & experimental systems

n/a	Involved in the study
☐	☒ Antibodies
☒	☐ Eukaryotic cell lines
☒	☐ Palaeontology and archaeology
☒	☐ Animals and other organisms
☒	☐ Clinical data
☒	☐ Dual use research of concern

Methods

n/a	Involved in the study
☒	☐ ChIP-seq
☐	☒ Flow cytometry
☒	☐ MRI-based neuroimaging

Antibodies

Antibodies used | Anti-phosphorylated-STAT5 (C11C5; Cell Signaling Technology #9359; 1:100 dilution), PE anti-CD19 (4G7; BD Biosciences #349209; 4uL/10^6 cells), APC-eFluor 780 anti-CD34 (4H11; eBioscience #47-0349-42; 4uL/10^6 cells), eFluor 450 anti-CD45 (2D1; eBioscience #48-9459-42; 5uL/10^6 cells), Super Bright 645 anti-CD3 (OKT3; eBioscience #64-0037-42; 3.5uL/10^6 cells), PerCP-eFluor710 anti-CD34 (4H11; eBioscience #46-0349-42; 4uL/10^6 cells), PE-Cy7 anti-CD38 (HIT2; BD Biosciences #560677; 3.5uL/10^6 cells), APC anti-CD90 (5E10; BD Biosciences #559869; 5uL/10^6 cells), FITC anti-CD45RA (HI100; BD Biosciences #555488; 5uL/10^6 cells), PE-Cy5 anti-CD49f (GoH3; BD Biosciences #551129; 4.5uL/10^6 cells), AlexaFluor700 anti-CD10 (CB-CALLA; eBioscience #56-0106-42; 5uL/10^6 cells), biotin anti-FLT3 (4G8; custom conjugation from BD Biosciences; 8uL/10^6 cells) and Qdot605 streptavidin (Thermo Fisher Scientific #Q10101MP; 3uL/10^6 cells)

Validation | We used commercially available antibodies from Cell Signaling Technology, BD Biosciences, and Thermo Fisher Scientific that were validated to bind human antigen targets.

Flow Cytometry

Plots

Confirm that:

☒ The axis labels state the marker and fluorochrome used (e.g. CD4-FITC).

☒ The axis scales are clearly visible. Include numbers along axes only for bottom left plot of group (a 'group' is an analysis of identical markers).

☒ All plots are contour plots with outliers or pseudocolor plots.

☒ A numerical value for number of cells or percentage (with statistics) is provided.

Methodology

Sample preparation | Samples consisted of primary human leukemia biopsies from peripheral blood (n=85) and bone marrow (n=8). Samples were thawed, labelled with antibodies, and flow-sorted or separated via magnetic beads.

Instrument | BD FACSAria III

Software | BD FACS Diva was used for cell sorting and BD FlowJo was used for post-sort analysis.

Cell population abundance | 5~10 million cells from bulk leukemia biopsy samples were used for flow sorting. Leukemic blasts accounted for >70% in most cases.

Gating strategy | 1) FSC-A vs. SSC-A (both medium to high); 2) FSC-H vs. FSC-W (FSC-W low); 3) SSC-H vs. SSC-W (SSC-W low); 4) FSC-A vs. PI (PI low); 5) CD34, CD19, and sometimes CD45 were used to identify and sort blasts.

☒ Tick this box to confirm that a figure exemplifying the gating strategy is provided in the Supplementary Information.
